# Predicting RNA Structure Utilizing Attention from Pretrained Language Models

**DOI:** 10.1021/acs.jcim.4c02094

**Published:** 2025-07-02

**Authors:** Ioannis Papazoglou, Alexios Chatzigoulas, George Tsekenis, Zoe Cournia

**Affiliations:** † 89223 Biomedical Research Foundation, Academy of Athens, Athens 11527, Greece; ‡ Department of Biology, National and Kapodistrian University of Athens, Athens 15784, Greece

## Abstract

RNA possesses functional significance that extends beyond the transport of genetic information. The functional roles of noncoding RNA can be mediated through their tertiary and secondary structure, and thus, predicting RNA structure holds great promise for unleashing their applications in diagnostics and therapeutics. However, predicting the three-dimensional (3D) structure of RNA remains challenging. Applying artificial intelligence techniques in the context of natural language processing and large language models (LLMs) could incorporate evolutionary information to RNA 3D structure predictions and address both resource and data scarcity limitations. This approach could achieve faster inference times, while keeping similar accuracy outcomes compared to employing time-consuming multiple sequence alignment schemes, akin to its successful application in protein structure prediction. Herein, we evaluate the suitability of currently available pretrained nucleic acid language models (RNABERT, ERNIE-RNA, RNA Foundational Model (RNA-FM), RiboNucleic Acid Language Model (RiNALMo), and DNABERT) to predict secondary and tertiary RNA structures. We demonstrate that current nucleic acid language models do not effectively capture structural information, mainly due to architectural constraints.

## Graphical abstract

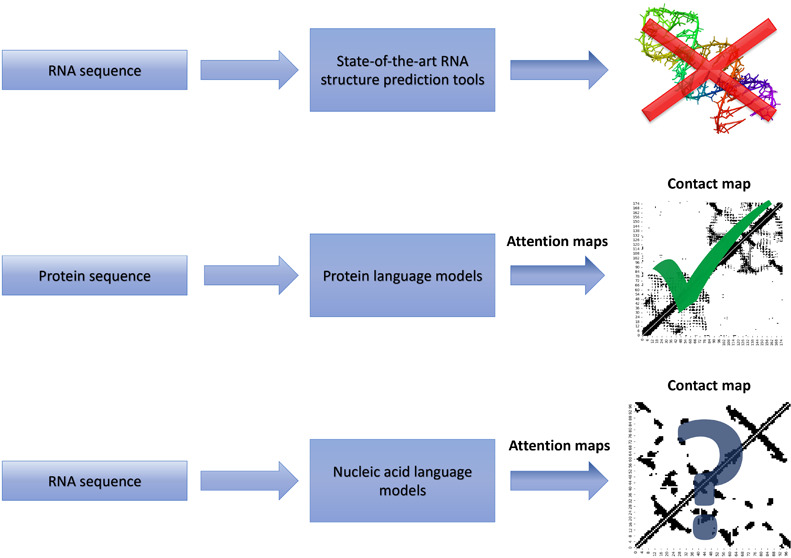

## Introduction

RNA constitutes a diverse group of molecules transcribed from specific DNA regions and is important not only in carrying genetic information (mRNA) but also in regulating gene expression (noncoding RNA, ncRNA) and other functions such as participating in enzymatic catalysis.^1,2^ However, unlike the simple linear molecule of the messenger mRNA, ncRNAs (hereafter referred to as “RNA” for simplicity) adopt highly structured shapes in space and interact with various molecules, including other nucleic acids, proteins, or small molecules. This inherent versatility enables them to effectively execute their regulatory functions, thus being equally crucial for life as proteins.^3,4^ Although recent progress in sequencing methods has led to the discovery of an increasing number of novel RNA sequences,^5^ experimental determination of RNA 3D structures is still far more limited compared to protein structures.^6^

This disparity in RNA experimental structure determination could potentially be addressed through structure prediction algorithms. Currently implemented, state-of-the-art Artificial Intelligence (AI) methods^7–11^ use structural information from evolution that is extracted from Multiple Sequence Alignments (MSA)^7^ or from structural fragment libraries,^8,9^ which offer a significant increase in structure prediction accuracy for well-annotated RNA; nevertheless, they still struggle with RNA sequences lacking sufficient homology data.^10–12^ Although recent advances in Artificial Intelligence (AI) have led to progress in RNA structure prediction, they have not yet achieved the same level of success seen in protein structure prediction.^13^ In particular, current AI methods for RNA structure prediction do not outperform the leading hybrid approaches such as AIchemy_RNA2,^14^ which integrate knowledge-based heuristics with energy-based principles, as shown in the latest CASP15 RNA structure prediction results.^15–78^ Unlike proteins, RNA sequences often lack sufficient homologous sequences to construct high-quality MSAs, particularly for novel RNAs, limiting the effectiveness of AI-driven predictions.^16^

Meanwhile, the protein three-dimensional (3D) structure prediction field embraced the use of natural language processing (NLP) techniques as an alternative, faster means of obtaining the evolutionary information needed for 3D structure predictions. Large protein language models (pLMs) that are pretrained on extensive unlabeled sequence data, can uncover contextual information encoded in the amino acid language, including structural features.^17^ The structural information is stored in the weights of the multihead self-attention mechanism, which, after processing a query sequence, generates attention maps that directly project amino acid interactions within the protein 3D structure, akin to a contact map.^18,19^ Based on this concept, Meta AI developed the ESMFold framework,^20^ and achieved accuracy comparable to the MSA-dependent AlphaFold2 ^21^ and RoseTTAFold,^22^ while being 6–60 times faster depending on the query sequence length.^20^

NLP and language models (LMs) have also been applied in RNA research. A recent approach, RNA-MSM, is an MSA-based RNA language model,^23^ which demonstrates enhanced capabilities in extracting biologically meaningful relationships using unsupervised learning and can directly reveal RNA secondary contacts through its attention maps. Nevertheless, the dependence of this model on MSAs results in extended prediction times. For instance, obtaining alignments for a single RNA sequence consisting of 60 nucleic acids reportedly requires an average of 9 h.^23^

Several pretrained nucleic acid LMs have been developed to process individual RNA sequences and generate informative embeddings, thereby eliminating the need for MSA in 3D structure prediction. These models include RNABERT,^24^ the RNA Foundational Model (RNA-FM),^25^ ERNIE-RNA,^26^ and the RiboNucleic Acid Language Model (RiNALMo).^27^ Notably, RNA-FM was developed alongside RhoFold+,^25^ a transformer-based model, that employs the same principles as AlphaFold2, and utilizes evolutionary information from the pretrained LM to predict atomic coordinates. The application of RNA-FM in RhoFold+ has shown promising performance in predicting RNA 3D structures, as demonstrated in CASP15 and RNA-Puzzles targets;^11,25,28–31^ however, its performance is primarily limited to naturally-occuring RNA sequences and declines significantly with synthetic or engineered constructs. RNABERT, ERNIE-RNA, and RiNALMo also demonstrate contextual learning, such as recovering the likelihood of canonical Watson–Crick or noncanonical base pairs based on the query sequence context, and show enhanced performance in downstream structural predictions when their output embeddings are utilized.^24–27^ However, the potential of their attention mechanisms to directly uncover molecular structures remains largely unexplored. While LMs primarily derive their understanding from the weights of the feed-forward layers,^32,33^ existing pretrained nucleic acid LMs are designed to incorporate structural or contextual information into their output embeddings.

In this work, we investigate whether the intermediate attention mechanisms of current nucleic acid language models can directly project the secondary and tertiary contacts defining the RNA structure, similar to what has been accomplished with pLMs. By leveraging this potential capability, structural information could be extracted by employing a simple machine learning (ML) classifier. Such a classifier would require minimal training structural data within a few-shot learning framework, tackling data scarcity and boosting inference speed. Here, we first validate the contact prediction approach for proteins using LM attention by reimplementing it for three pLMs: ESM2–3B,^20^ ProtT5-uniref50XL,^34^ and ProtT5-bfdXL^34^ (Table 1) using ∼15,000 protein structures (Figures S1 and S2A). Building on these predictions, we extend the methodology to predict RNA tertiary and secondary structure contacts, represented as contact maps using the attention of pretrained nucleic acid LMs. We evaluate four RNA-specific LMs: RNABERT, ERNIE-RNA, RNA-FM, and RiNALMo (Table 1) using ∼25,000 RNA secondary and 425 RNA tertiary structures (Figures S1 and S2B,C). We also examine a DNA-specific LM, DNABERT^35^ (Table 1), to explore its suitability for RNA structure prediction assuming RNA-DNA coevolution. Finally, we benchmark our trained classifier models against current state-of-the-art tools for RNA structure prediction, specifically trRosettaRNA,^36^ AlphaFold3,^37^ RhoFold+,^25^ DeepFoldRNA,^38^ and FarFar2/ARES^39,40^ for tertiary structure and SpotRNA,^41^ RNAFold,^42^ mxFold2,^43^ and RNA-MSM^23^ for secondary structure prediction. An overview of this procedure is provided in Figure S1 of the Supporting Information (SI).

**Table 1:** Available Biological LMs and a Comparison of Their Features

language model	parameters	pretraining	architecture	attention	seq length
ESM2-3B^20^	3B	60M seqs	BERT	36 × 40	1024+
ESM2-15B^20^	15B			48 × 40	
ProtT5-uniref50XL^34^	3B	45M seqs	T5	24 × 32	512+
ProtT5-uniref50XXL^34^	11B			24 × 128	
ProtT5-bfdXL^34^	3B	2.1B seqs	T5	24 × 32	512+
ProtT5-bfdXXL^34^	11B			24 × 128	
RNABERT^24^	480K	76K seqs	BERT	6 × 12	440
ERNIE-RNA^26^	86M	23M seqs	ERNIE	13 × 12	512+
RNA-FM^54^	100M	23M seqs	BERT	12 × 20	1024+
RiNALMo^27^	650M	36M seqs	BERT	33 × 20	1024+
RNA-MSM^23^	96M	4069 fams	BERT	10 × 12	512 (MSA)
UNI-RNA^55^	400M	1B seqs	BERT	24 × 20	1280+
DNABERT-3 ^35^	86M	3.2B nts	BERT	12 × 12	512

## Methods

### Data Set Generation and Preprocessing

We curated and constructed three different data sets: (1) a protein tertiary structure data set to use for the methodology validation, (2) an RNA tertiary (3D) structure data set, and (3) an RNA secondary (two-dimensional (2D)) structure data set for the redevelopment of the methodology for RNA structure prediction.

The protein data set was built by utilizing the trRosetta data set,^44^ which contains experimentally resolved 3D protein structures sourced from the Protein Data Bank (PDB). The trRosetta data set had already undergone redundancy reduction by its creators, and thus, we maintained its integrity. Our only modification was the exclusion of proteins with more than 1024 amino acids due to memory limitations in our GPU resources (24 GB of VRAM). Approximately 15,000 protein tertiary structures remained in the data set (Figure S2A).

For the RNA tertiary structure data set, we initially assessed the PDB^45^ and the Nucleic Acid knowledgebase (NAKB)^6^ to gather all available RNA with experimentally resolved 3D structures. We removed lower-quality data containing unmodeled residues in their structure or modified/unusual bases in their sequence, and eliminated the molecules with sequence lengths greater than 1024 nucleotides, which was the maximum token input size we could process, taking into consideration our computational resources. Then, we performed redundancy reduction using the CD-HIT-EST^46^ algorithm (*sequence identity threshold* set to 0.8, *length difference cutoff* set to 0.9), resulting in a final data set of 425 molecules with their tertiary structures (Figure S2B).

For the RNA secondary structure data set, we merged three publicly available secondary structure collections: bpRNA,^47^ RNAStralign,^48^ and ArchiveII.^49^ While we acknowledge that these data sets have lower-quality and computationally derived annotations compared to the experimentally resolved structures in PDB and NAKB, their large size, and diversity provide a broader training set for AI models. Moreover, they have been successfully employed in other studies to train robust frameworks,^41,43,50^ demonstrating their practical utility in effectively generalizing structural patterns, despite their limitations. To further ensure consistency, we preprocessed the data sets by cleaning and reducing redundancy as described for the RNA tertiary structure data set, resulting in a final data set of approximately 30,000 molecules with their corresponding secondary structures (Figure S2C).

The final step was to represent each molecular structure in binary contact maps (Figure S3). To generate the binary contact maps for tertiary structure data, we computed the distances between atom pairs and created binary contact maps with a 9.5 Å cutoff distance. The cutoff value was selected after evaluating 8 9.5, and 12 Å as cutoffs by assessing the structural index across all of the LMs studied. Our analysis revealed that the 9.5 Å cutoff has an optimal balance between specificity and sensitivity, achieving an informative representation of structure by avoiding either too generalized or overly specific contact maps (Figure S4). To retrieve contact maps for RNA secondary structure, we converted the dot-bracket notation into binary contact maps (Figures S5 and S6). Finally, we excluded the close contacts within a range of 6 positions between amino acids and 4 positions between nucleotides from the generated contact maps. Excluding these nearby contacts, which represent the primary structure known to the LMs (sequence of bases), ensured that the classifier would focus on learning long-range interactions and distinguishing structural information relevant to the more complex secondary and tertiary levels during the structure prediction task.

More information about data collection and preprocessing and the generation of contact maps is provided in SI Sections 2 and 3.

### Language Model Selection—Attention Mechanism Assessment

For generating protein contact maps in the context of pLMs for the validation of our methodology, we used the Evolutionary Scale Model^20^ in the version of 3 billion parameters (ESM2–3B) and additionally, the ProtTrans model^34^ versions uniref50XL (ProtT5-uniref50XL)^51^ and bfd-XL (ProtT5-bfdXL).^52,53^ For generating RNA contact maps, we examined the four pretrained nucleic acid LMs that are currently available, namely, RNABERT,^24^ RNA-FM,^54^ ERNIE-RNA,^26^ and RiNALMo,^27^ with all processing a single query sequence as input. Additionally, we independently tested DNABERT-3,^35^ assuming significant shared similarities between DNA and RNA languages due to their coevolution. However, DNABERT-3 was not included in direct comparisons with the other models due to differences in their foundational approaches (specifically, the utilization of different ground truth, which is 3mer contact maps, as detailed in SI Section 3 and Figure S5).

In our experiments, we did not assess RNA-MSM,^23^ which accepts an MSA input rather than individual query sequences, retaining the inference-speed limitations because it additionally requires computationally intensive MSA generation for each query. Nevertheless, because its attention mechanism has been shown by its developers to uncover secondary structure, we included RNA-MSM in our benchmark of state-of-the-art RNA structure prediction methods to evaluate its knowledge alongside the other LMs to ensure completeness of our study. Additionally, another available RNA LM: UNI-RNA,^55^ was excluded from our assessments due to the current unavailability of its source code and pretrained weights. Table 1 provides a summary of these LMs, while additional details about their inference can be found in Section 4 of the SI.

To assess whether the selected LMs have captured evolutionary structural information in their attention mechanism, we used the mathematical equation described in ref 19:

1 $$p\left(f\right)=\frac{\sum_{x\in X}\sum_{i=1}^{\left|x\right|}\sum_{j=1}^{\left|x\right|}f\left(i,j\right)\cdot l_{a_{i,j}>\theta }}{\sum_{x\in X}\sum_{i=1}^{\left|x\right|}\sum_{j=1}^{\left|x\right|}l_{a_{i,j}>\theta }}$$

and

2 $$f\left(i,j\right)=\{\begin{array}{ll} 1, & if\;\left(i,j\right)\;is\;contact\\ 0, & if\;not \end{array}$$

3 $$l_{a_{i,j}}=\{\begin{array}{ll} a_{i,j}, & if\;\theta \;and\;a_{i,j}>\theta \\ 0, & if\;\theta \;and\;a_{i,j}\leq \theta \end{array}$$

where the indicator function *f*(*i*,*j*) processes the residue pairs inside the target contact maps and returns 1 if the token pair (*i*,*j*) is a contact. The $l\alpha_{i,j}$ term runs inside the extracted attention maps and recalls the value *a*_*i*,*j*_ inside the token pair (*i*,*j*), and based on a predefined threshold θ, it returns the attention weight to the calculation. Thus, the θ factor serves as a threshold for selecting attention weights that correspond to contacts with high confidence.

The literature suggests conducting the mathematical analysis using a threshold (θ) value of 0.3;^19^ however, an alternative equation eliminating this threshold (θ) is described in Appendix B1: “Alternative Attention Agreement Metric” in ref 19:

4 $$p\left(f\right)=\frac{\sum_{x\in X}\sum_{i=1}^{\left|x\right|}\sum_{j=1}^{\left|x\right|}f\left(i,j\right)\cdot a_{i,j}\left(x\right)}{\sum_{x\in X}\sum_{i=1}^{\left|x\right|}\sum_{j=1}^{\left|x\right|}a_{i,j}\left(x\right)}$$

Finally, in any thresholding case, the calculated *p*(*f*) represents the percentage of attention weights (*a*) that indicate the pairing of structural tokens within each attention head of the architecture:

5 $$p\left(f\right)=\frac{\sum [\mathrm{all}\;a\;(>\theta )\;\mathrm{indicating}\;\mathrm{structural}\;\mathrm{token}\;\mathrm{pairs}]}{\sum [\mathrm{all}\;a\;(>\theta )]}$$

### Feature Extraction Using Attention Maps

For feature extraction, we processed each RNA sequence through the respective LM and obtained attention maps with the self-attention weights. We then subjected these attention maps to the symmetrization operation to transform them into symmetrical representations and Average Product Correction (APC)^56^ to refine the attention weights by eliminating the general trends of attention over the token pairs (more information in SI Section 5). Subsequently, we treated each token pair as an individual sample, utilizing all pairwise attention weights from the maps as features. Furthermore, we associated each token pair feature with the corresponding binary value from the contact map, which served as the target variable (Figure S7).

Finally, the generated data sets presented a major imbalance ratio, which we addressed by under-sampling the majority class utilizing the imbalanced-learn Python toolbox,^57^ thus ensuring that during training we used an equal number of samples for each class. The constructed data sets were used for training the classifiers to predict token pairs in contact maps. More information about the overall process is provided in SI Section 5.

### Training Machine Learning Classifiers for Protein and RNA Structure Prediction

For protein structure prediction, we generated a training data set of 20 randomly selected proteins from the trRosetta structure collection and used the rest of the 14,988 proteins as test instances to evaluate the predictions.

For RNA tertiary and secondary structure prediction, the collected molecules were initially split into an 85–15% training-to-test ratio, resulting in 362 training and 63 test instances used for tertiary structure predictions, and 25,715 training and 4,537 test instances for secondary structure prediction. Additionally, within each RNA training set, we further divided the molecules into 5 training batches (Figure S8). These batches spanned from 20 individual molecules to the entire training set, allowing us to explore learning convergence through portions of the available data, as we intended to identify the optimal number of molecules required for training to yield favorable results. Eventually, the predicted contact maps were flattened to one-dimensional (1D) binary vectors and compared against the original target contact maps using the *F*_1_ score and Matthew’s Correlation Coefficient (MCC) metrics. Subsequently, we computed the mathematical average of these individual scores to assess the overall performance of each classifier. Detailed information about the batch training and evaluation is provided in SI Section 6.

For the protein data set, we only trained a logistic regression classifier^58^ from the sci-kit library,^59^ while for the RNA tertiary and secondary structure data sets, we trained five classifiers: logistic regression,^58^ decision tree,^60^ random forest,^61^ and a multilayer perceptron^62^ from the sci-kit library,^59^ and the XGBoost^63^ classifier (Table S1). For the vast amount of RNA secondary structure data, we also trained a Convolutional Neural Network (CNN)^64^ using PyTorch^65^ (Figure S9 and Table S2). More information is provided in SI Section 6.

### Comparison of the Developed Classifier Models with State-of-the-Art Models

To assess the prediction efficacy of the developed classifier models that we developed, we compared them against established state-of-the-art methods, namely, trRosettaRNA,^36^ AlphaFold3,^37^ RhoFold+,^25^ DeepFoldRNA,^38^ and FarFar2/ARES^39,40^ for tertiary structure and against the established secondary structure prediction tools SpotRNA,^41^ RNAFold,^42^ mxFold2,^43^ and RNA-MSM.^23^

For this evaluation, we utilized an additional data set comprising 33 RNA molecules with experimentally resolved structures sourced from the PDB. These molecules were selected based on publication date criteria (June 2022 and onward) and a sequence length shorter than 100 nucleotides. The publication date filter ensured that these molecules were not utilized in training the AI components of other state-of-the-art methods, thus remaining largely unseen. Furthermore, we excluded any structures similar to those in our training data sets used for classifier development by utilizing CD-HIT-EST (*sequence identity threshold* set to 0.8, *length difference cutoff* set to 0.9). Additionally, we identified and reported whether there are any closely related RNA molecules with known structures in the PDB that could potentially bias the prediction process (Table S3).

The predicted tertiary and secondary structures were converted into contact maps and compared against the predictions from our developed classifiers trained on nucleic acid LM attention features. Additionally, for a comprehensive benchmark, the predicted atomic coordinates were aligned with the original structures, and the prediction accuracy was evaluated using Root Mean Square Deviation (RMSD) and Template Modeling (TM) scores (more information is provided in SI Section 8).

## Results

### Methodology Validation in Protein Tertiary Structure Prediction

To illustrate the efficacy of pLM attention in deducing protein structure, Figure 1 presents a selected attention map extracted from ESM2–3B after processing the lysozyme sequence. We quantified the alignment of attention and structural information using eq 1 for LM assessment (θ = 0.3) over the curated data set of 15,008 proteins, revealing that all examined pLMs, regardless of their architecture type (BERT or T5), have uncovered structure in their self-attention layers (Figure 2). These findings are consistent with the existing literature, which suggests that more complex information, such as structure, tends to be captured in the upper layers and heads of the attention mechanism.^19^ Remarkably, some of the top layers and heads exhibited a percentage of up to 95.8% (ESM2–3B), 93.1% (ProtT5-uniref50XL), and 87.7% (ProtT5-bfdXL) of attention weights aligning with structural contact pairs. Notably, these pLMs were exclusively trained on sequence data without any prior exposure to structural data, making the prevalence of such structurally aware attention heads an intriguing phenomenon.

**Figure 1:**
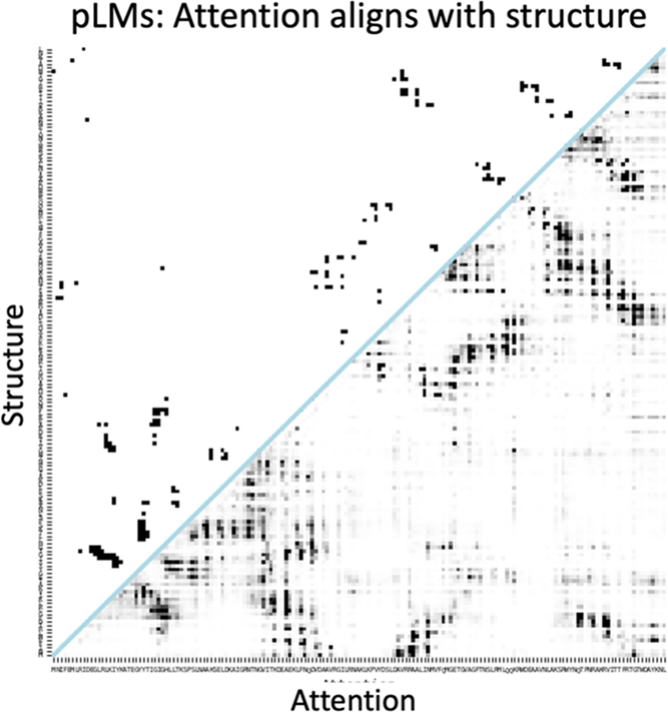
pLM attention (below the blue line) can generate protein contact maps (above the blue line). Attention was extracted from the 8th head of the 35th layer of ESM2–3B after processing the lysozyme sequence (UniProt ID: P00720). The contact map (above the blue line) was generated from resolved 3D coordinates of the protein in PDB (code: 253L^66^).

**Figure 2:**
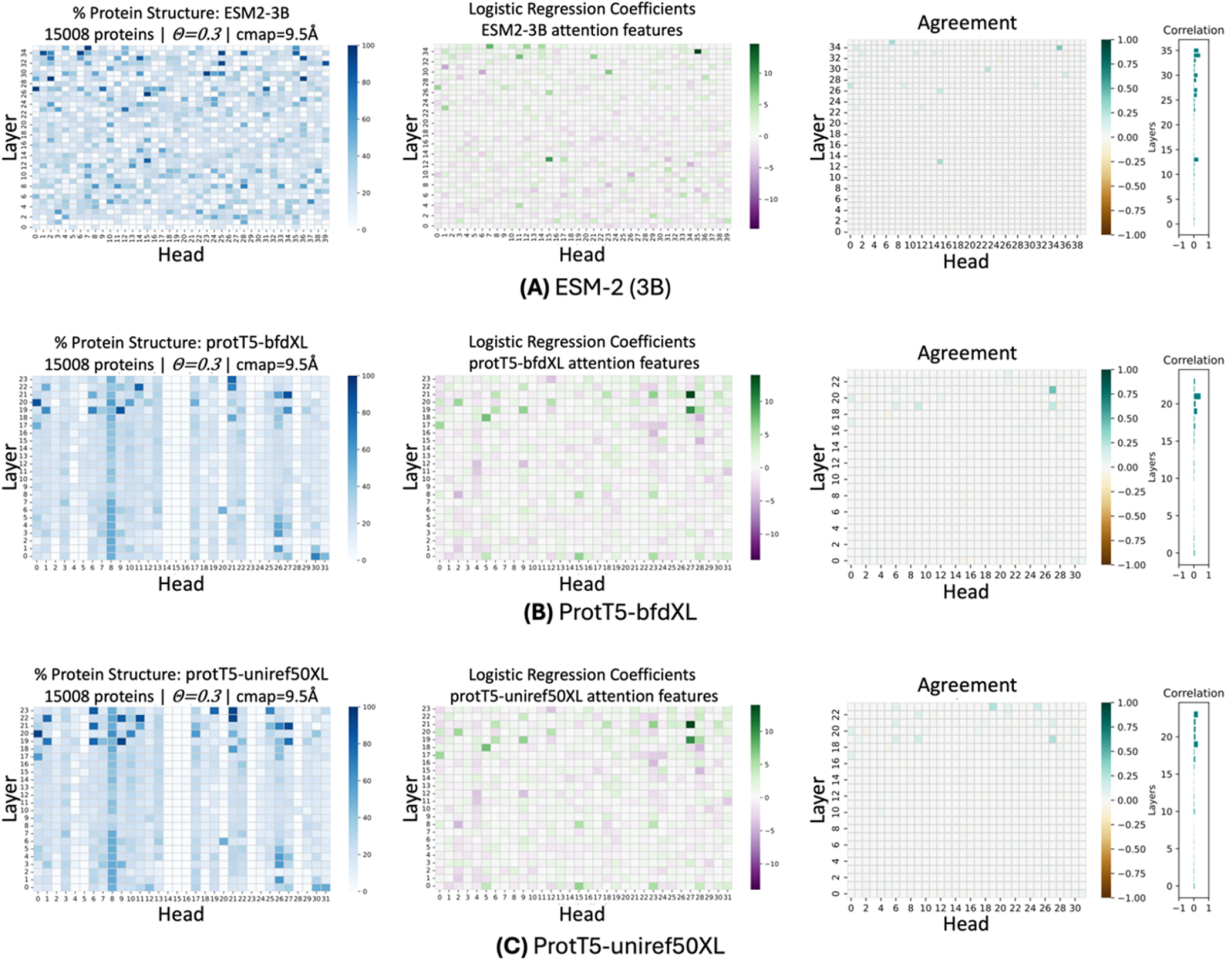
Comparison of pLM attention capacity to capture protein structural information: (A) ESM2–3B, (B) ProtT5-uniref50XL, and (C) ProtT5-bfdXL. The heatmaps illustrate the attention architecture of each pLM, with layers represented on the *y*-axis and attention heads on the *x*-axis. The “% Protein Structure” plots quantify the structural information captured by each attention head, estimated using eq 1 on 15,008 proteins (θ = 0.3). The “Logistic Regression Coefficients” plots highlight the attention heads identified as most informative by logistic regression, trained on a data set of 20 randomly selected proteins. The extent of structural information distribution among attention heads, which was mathematically estimated, agrees with the attention heads identified as most informative by logistic regression, as seen in the “Agreement” plots.

After evaluating the logistic regression classifiers, which were trained on a randomly selected subset of just 20 proteins using pLM attention, their performance on the contact prediction downstream task was evaluated on the remaining 14,988 proteins from the data set. The average obtained *F*_1_ scores for classifiers trained on features extracted from ESM2, ProtT5-uniref50XL, and ProtT5-bfdXL attention were 0.81, 0.79, and 0.76, respectively (Figures 3A and S4). Also, when visually assessing the predictions of these logistic regression classifiers, we observed that they do indeed predict contact maps with highly refined structural information (Figure 3B). Noteworthy, these findings refer to the second largest versions that each pLM offers, which we were restricted to using due to resource limitations. Nevertheless, Meta AI suggests that their largest developed architecture yield even better predictions.^20^

**Figure 3:**
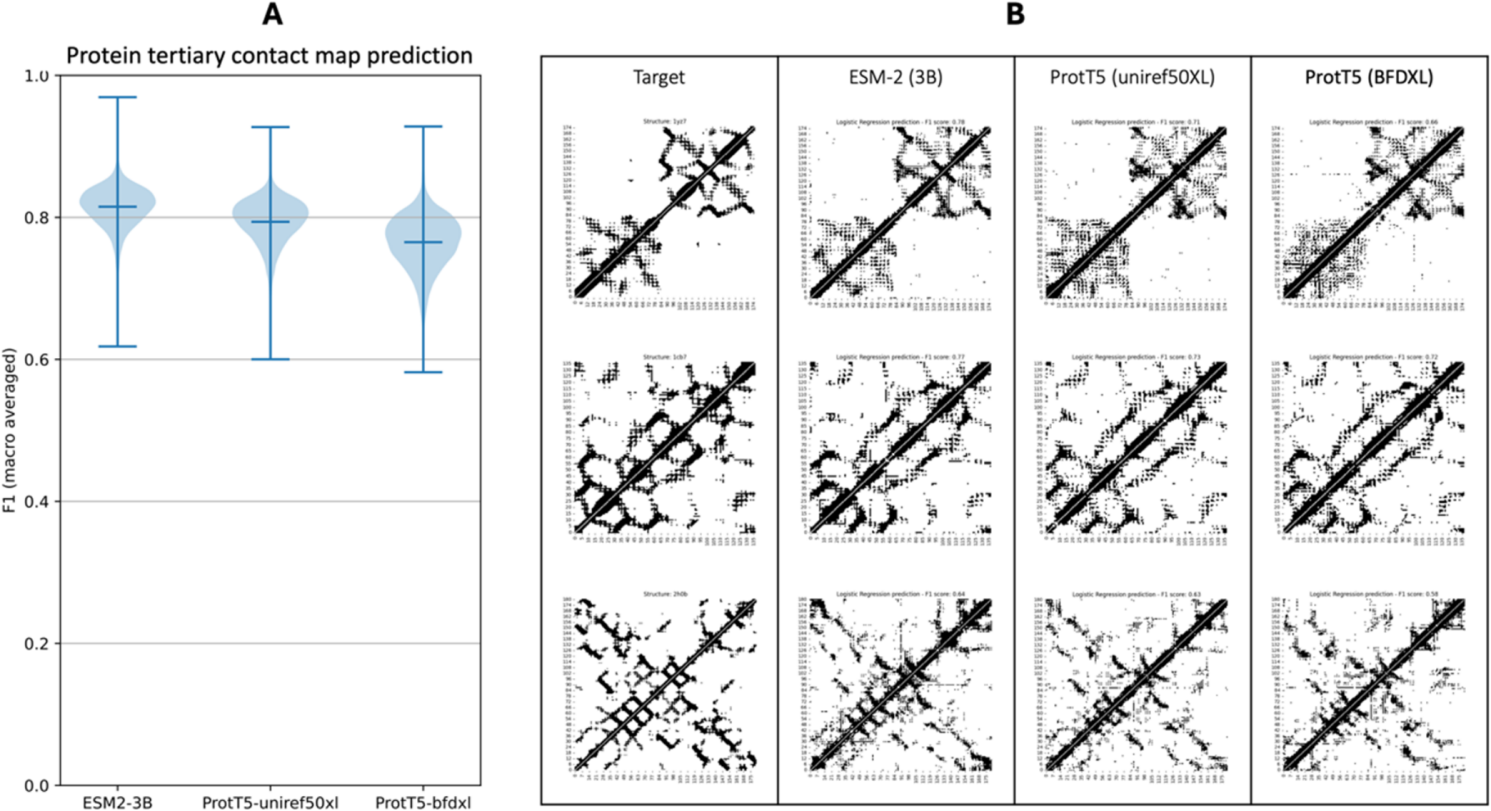
(A) *F*_1_ score evaluation in 14,988 proteins (test set). Predictions were made using a logistic regression classifier trained on the primary sequence of just 20 proteins and utilizing attention features from ESM2–3B, ProtT5-uniref50XL, and ProtT5-BFDXL pLMs. (B) Predicted contact maps using each of the three pLM attention; PDB codes 1YZ7,^67^ 1CB7,^68^ 2H0B^69^ (top to bottom).

Upon revisiting and analyzing the logistic regression coefficients, we observed another significant finding: the learned weights consistently assigned greater importance to specific architectural layers and heads identified in our mathematical estimation (see Figure 2). This alignment between our mathematical estimation and the classifier’s findings serves as a robust validation of our LM selection methodology.

### Methodology Reimplementation for RNA Tertiary Structure Prediction

Quantitative evaluation of the nucleic acid LMs attention mechanisms revealed varying degrees of tertiary structural information in their multihead attention mechanisms. Specifically, RNABERT and ERNIE-RNA demonstrated attention weights percentages corresponding to structure of up to 13.8 and 10.7%, respectively. In constrast, RNA-FM and RiNALMo exhibited heads with percentages of attention weights aligning with structural nucleotide pairs of up to 55 and 69.1%, respectively (Figure 4A–D).

**Figure 4:**
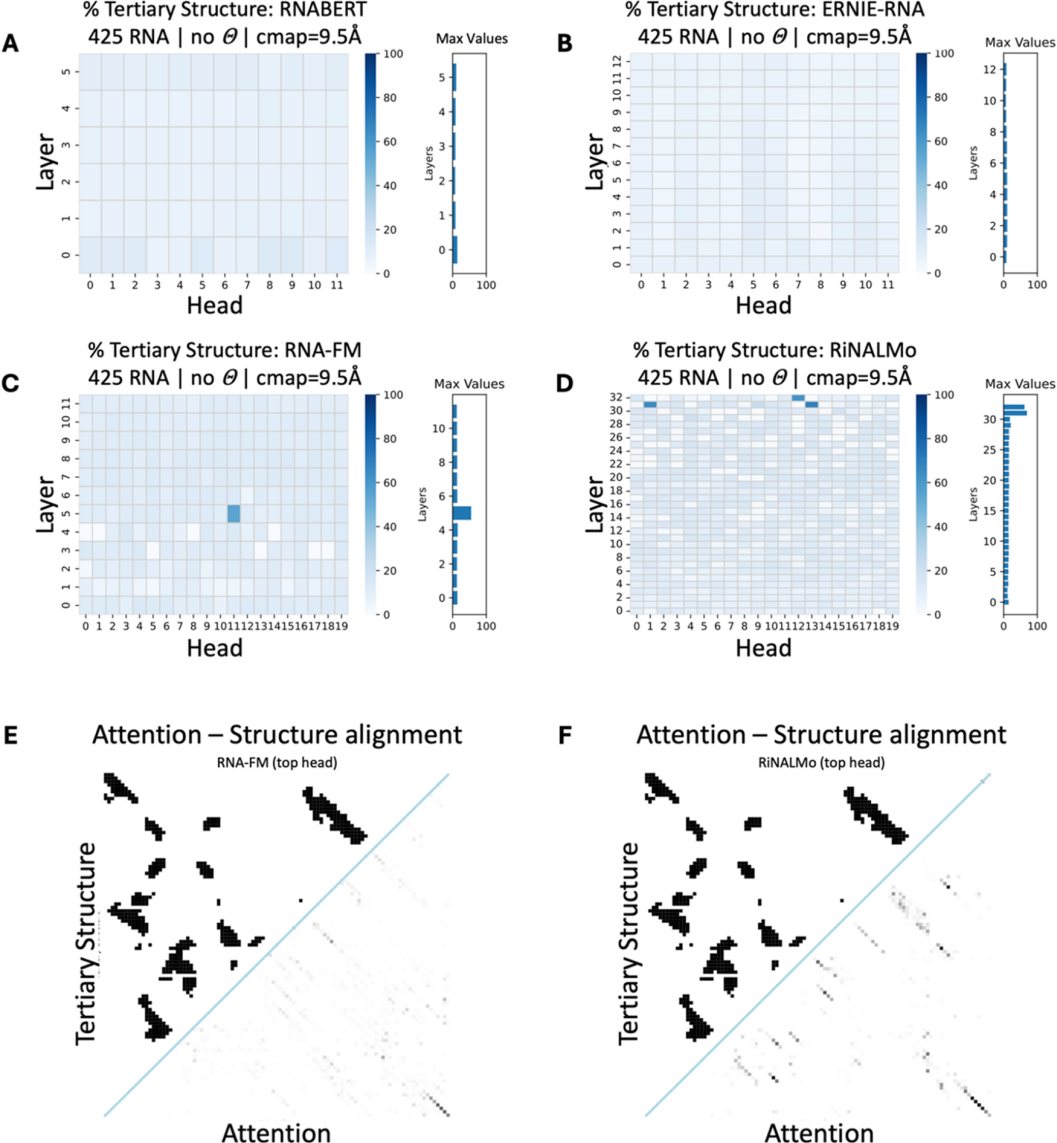
Percentage of secondary structure information in the different nucleic acid LMs. (A, B) RNABERT and ERNIE-RNA show limited structural information across all attention heads because top-scoring heads are estimated to contain 13.8 and 10.7% of attention aligned with structure, respectively. In contrast, (C, D) RNA-FM and RiNALMo display structural alignment, particularly in intermediate and higher layers (55 and 69.1% of attention heads aligned with the structure, respectively). (E, F) The top-scoring attention maps from RNA-FM and RiNALMo demonstrate structural alignment with resolved 3D tertiary structures. The layout visually compares the structural alignment between the model’s attention (below the line) and the true RNA tertiary contact map (above the line), emphasizing how well the attention mechanism captures structural information. The contact map was generated using resolved 3D coordinates of RNA (PDB code: 4WFM^70^).

These estimations were calculated using the LM assessment in eq 4 without applying a threshold (θ). We noticed that thresholding significantly reduced the number of usable attention weights, eliminating most signals and leaving only highly localized patterns that fail to capture broader structural relationships (Figure S10). Instead, for nucleic acid LM evaluation we adopted the alternative eq 4 provided in ref 19. This approach allowed us to include all attention weights, both strong and weak, in our evaluation, enabling us to investigate whether even the smallest attention weights carry meaningful structural information. By avoiding thresholding, we ensured that nucleic acid LMs were evaluated comprehensively, reflecting their capacity to encode structural patterns (Figure S11).

When we visually inspected the attention mechanism after processing a single RNA molecule from PDB code: 4WFM^70^ from RNA-FM, and RiNALMo LMs, we observed no alignment between the attention of the top-scoring heads to the original RNA tertiary contact map (Figure 4E,F). However, the observed attention patterns align closely with the secondary structure of the molecule (see the next section). Upon this observation, we proceeded to ML training, hypothesizing that ML could uncover underlying, nonobvious relationships within the attention patterns that reflect structural information not directly visible through simple attention-structure alignment.

We trained the five ML classifiers on a training batch of 20 randomly selected RNA molecules using attention features from the various nucleic acid LMs. All classifiers showed similar performance in *F*_1_ macro averaged score and MCC score (Figure 5A, Tables S5 and S6). None of the classifiers could extract sufficient structural information from the attention features generated by the nucleic acid LMs, achieving poor performance when evaluated in the test set (up to a 0.53 average *F*_1_ score and 0.10 average MCC). This low performance further suggested that the attention mechanisms of the examined nucleic acid LMs did not learn descriptors of tertiary structure during their pretraining, which is crucial for this ML classification task.

**Figure 5:**
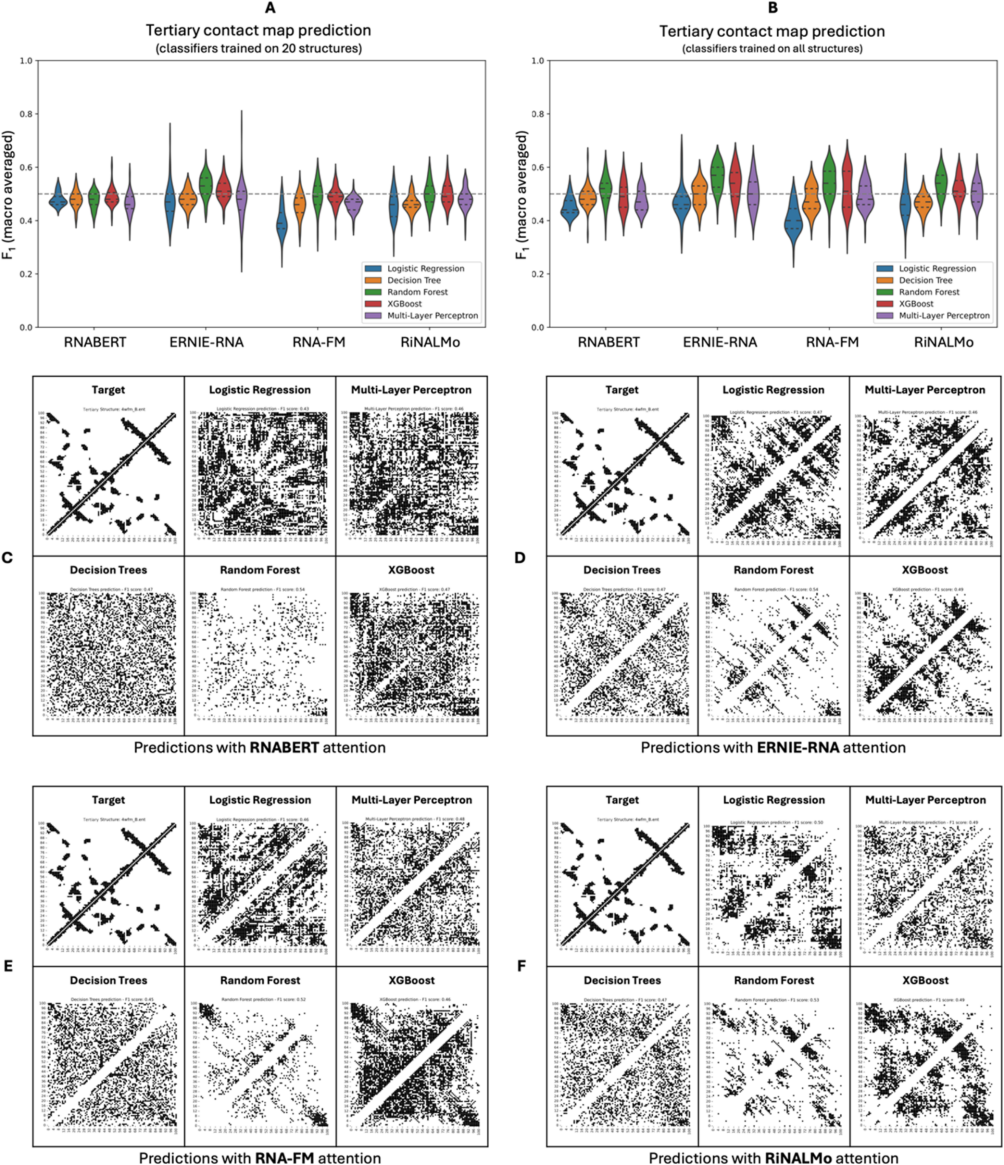
Evaluation of classifiers utilizing nucleic acid LM attention to predict RNA tertiary contact map prediction. (A) All five classifiers trained on 20 tertiary structures using attention features from each of the four RNA LMs performed poorly on the test set (*F*_1_ score ≤0.53). The random forest classifier consistently achieved the highest scores among them. (B) Retraining the classifiers using the full training data set resulted in similar performances, with the random forest classifier again outperforming others (*F*_1_ score ≤0.56). (C–F) Visualization of the predicted tertiary contact maps for each ML classifier (trained on the full data set) using RNA LM attention explains the poor evaluation metrics values. Even when correct contacts were predicted, they were obscured by considerable noise from false positives, making interpretation challenging. (Example RNA PDB code: 4WFM^70^).

Even after retraining the classifiers using the larger batches or the entire training set, their performance remained inadequate (Figures S12–S13 and Tables S5–S6). The random forest classifier trained on the full data set showed the best performance among all classifiers, when evaluated in the test set (0.56 average *F*_1_ score, 0.17 average MCC, Figure 5B), although even this score suggests that the predictions are close to random. Also, there was no significant improvement in performance across the different nucleic acid LMs used to generate attention features, which indicates that none of them managed to unsupervisingly capture structure during their pretraining phase. This overall lack of accuracy is further highlighted by visual inspection of individual predictions, which consistently showed unsatisfactory results regardless of the nucleic acid LM used each time (Figure 5C–E).

We recalculated the results using DNABERT-3, which also exhibited a small percentage of attention heads containing weights that align with structural nucleotide pairs (up to 15.5% alignment in the top-scoring heads; Figure 6A). After training the ML classifiers utilizing its attention, it was again the random forest classifier that performed better than others, though still not achieving high performance when evaluated on the test set. Results were consistent whether the classifiers were trained on 20 structures (0.46 average *F*_1_ score, −0.04 average MCC) or the entire training set (0.53 average *F*_1_ score, 0.09 average MCC) (Figures S10–S11 and Tables S5–S6). This unsatisfactory performance was also evident while visualizing the predictions; classifiers failed to establish any meaningful correlation between attention and structure (Figure 6B).

**Figure 6:**
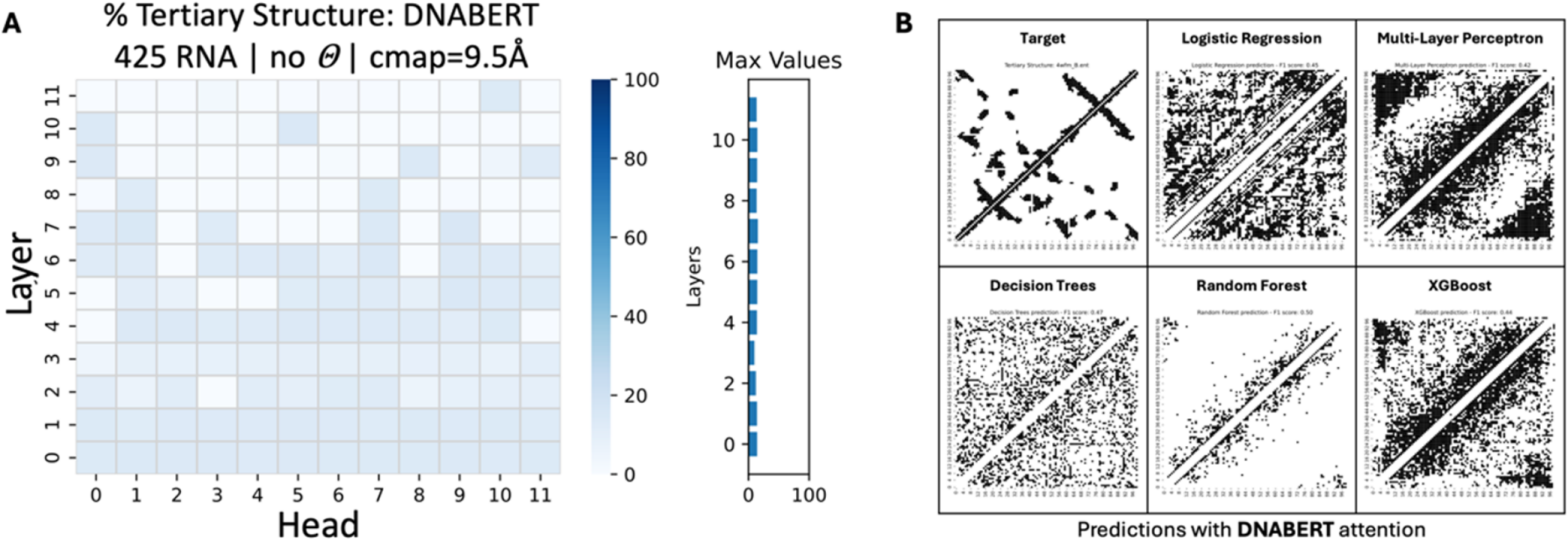
Assessment of DNABERT attention in RNA tertiary contact map prediction. (A) DNABERT failed to capture sufficient tertiary structural information after assessing over 425 tertiary structures, suggesting that its pretraining did not effectively captured RNA structure through its attention mechanism. (B) After visualizing the tertiary contact map predictions for each ML classifier (trained on the full data set), the random forest classifier exhibited the best performance. However, it only predicted short-distance contacts (close proximity contacts constrained by the primary sequence structure of bases—visualized around the main diagonal) (Example RNA PDB code: 4WFM^70^).

### Methodology Reimplementation for RNA Secondary Structure Prediction

Revising our methodology for the less complex task of secondary structure prediction, we assessed the percentage of attention weights per head aligned with the secondary structure (alternative LM assessment, eq 4, no threshold θ). Specifically, for RNABERT and ERNIE-RNA, we observed minimal alignment (up to 0.6 and 1.6% in the top heads, respectively). In contrast, the larger architectures of RNA-FM and RiNALMo exhibited a modest contextual alignment with secondary structural information in their attention mechanisms (up to 59.3 and 69.5% in the top heads, respectively; Figure 7). Interestingly, for these two nucleic acid LMs, the attention heads with the highest alignment percentage for the secondary structure were the same ones found to contain tertiary structural information. However, when visually inspecting RNA-FM and RiNALMo attention mechanisms after processing a single RNA molecule from PDB code: 4WFM,^70^ we did not observe alignment between the attention patterns and the secondary structure contact maps. In contrast, RNA-MSM, which is known to effectively encode secondary structural information within its attention weights,^23^ demonstrated clear attention-structure alignment in the top-scoring head (as reported in the literature) for this qualitative case study. Thus, RNA-MSM is currently the most effective LM capturing refined relationships between interacting base pairs at the secondary structural level, albeit its reliance on MSA generation introduces significant time constraints.

**Figure 7:**
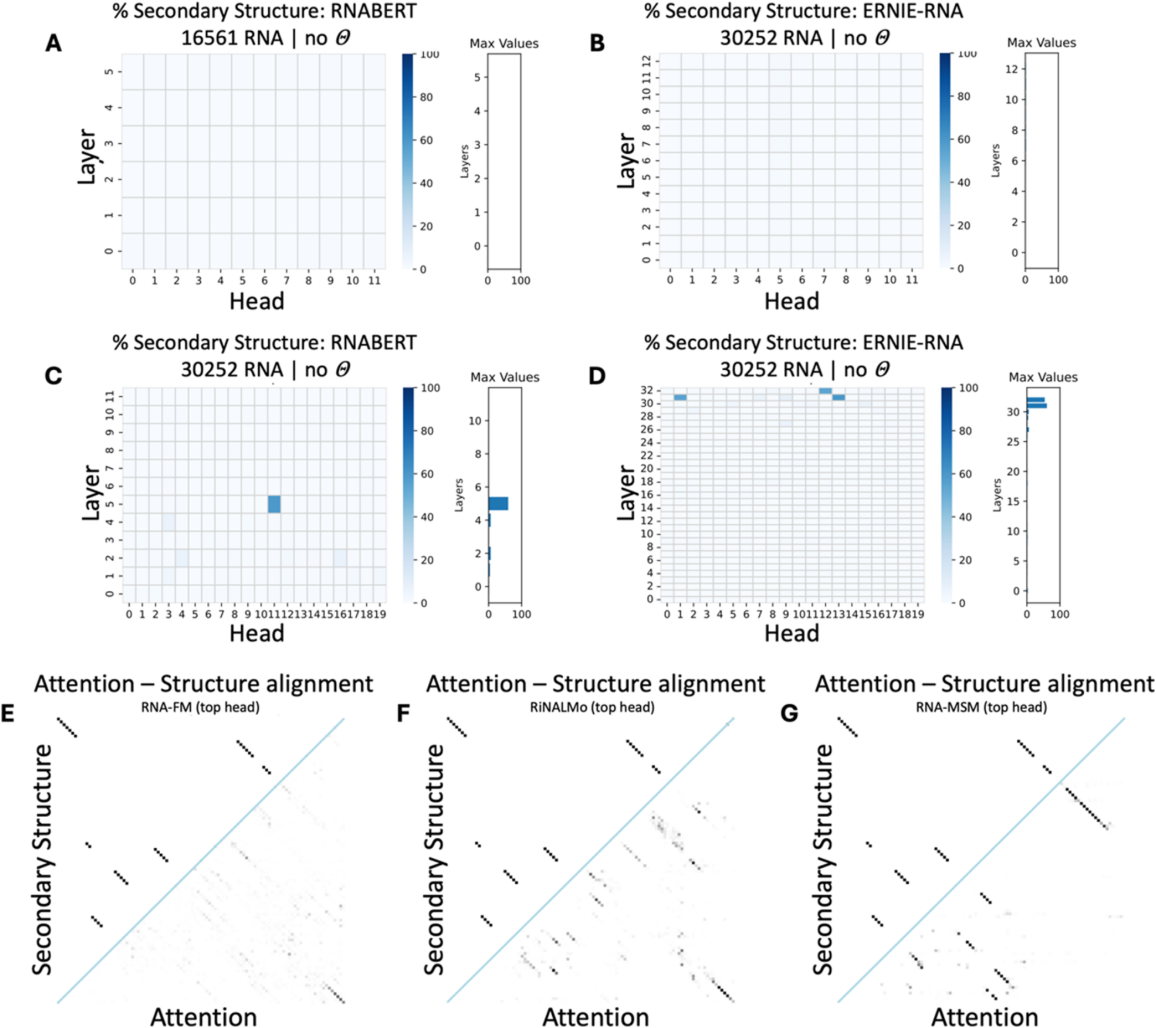
Percentage of secondary structure information on the different nucleic acid LMs. (A) RNABERT and (B) ERNIE-RNA did not capture enough structural information to predict RNA 3D structure, after estimating over 16,561 and 30,252 RNA secondary structures, respectively. (C) RNA-FM and (D) RiNALMo have little structural information in the intermediate or higher layers of their architecture, after estimating over 30,252 RNA secondary structures. (E) attention map from the top 6th head of the 12th layer of RNA-FM, (F) attention map from the top 32nd head of the 14th layer of RiNALMo, and (G) attention map from the 1st head of the 9th layer of RNA-MSM display how attention (bottom right) aligns with the RNA secondary structure (top left). The contact map was generated using the RNA molecule in PDB code: 4WFM.^70^

The ML classifier evaluation on the test set after training on the smaller batch of 20 randomly selected RNA molecules to predict their secondary structure indicated a trend where classifiers using attention features from the larger architecture RNA LMs showed slightly improved performance, although not significant (Figure 8A). Once again, the random forest emerged as the best-performing classifier across all evaluations. It achieved the following scores using attention from ERNIE-RNA (average *F*_1_ score of 0.58, average MCC of 0.27), RNA-FM (average *F*_1_ score of 0.55, average MCC of 0.19), and RiNALMo (0.57 average *F*_1_ score, 0.23 average MCC) (Tables S7 and S8).

**Figure 8:**
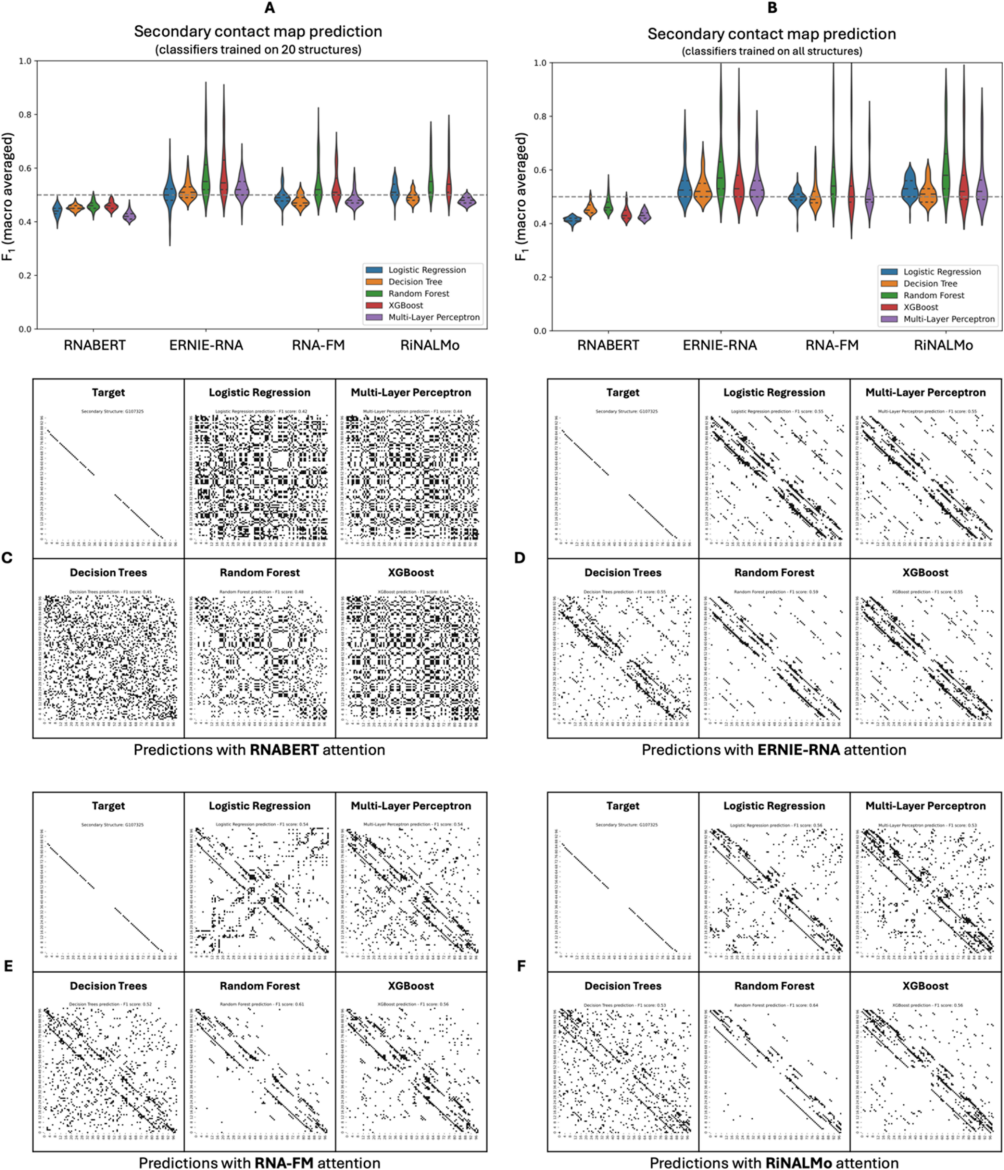
Evaluation of classifiers utilizing nucleic acid LM attention to predict RNA secondary contact map prediction. (A) The five ML classifiers using RNABERT attention (trained on 20 secondary structures) performed poorly (*F*_1_ score ≤0.46). Performance improved slightly when using the attention features from the other three larger RNA LMs, (*F*_1_ scores ≤0.59). Random forest and XGBoost classifiers consistently achieved the highest evaluation metrics. (B) The retrained on the full training data set ML classifiers performed similarly, with the random forest classifier once again emerging as the best one (*F*_1_ scores ≤0.60). (C–F) Visualizations of predicted secondary contact maps for ML classifiers trained on the full data set reveal predictions that successfully identify true positives (secondary contacts), alongside significant noise from false positive contact pairs. It is the random forest classifier using the largest available LM (RiNALMo) that eliminates at most the false positives in its predictions. (Example RNA molecule: “tdbD00010032”, source: RNAStralign^48^).

In contrast to the tertiary structure predictions, where performance remained mediocre even after training on the larger batches, the ML classifiers trained on the full training data set showed gradual improvement in predicting secondary structure (for a full analysis see Figures S12–S13 and Tables S7–S8). Again, the random forest classifier consistently outperformed the other classifiers, and more specifically, when using attention from ERNIE-RNA, it achieved an average *F*_1_ score of 0.60 and average MCC of 0.32; with RNA-FM, it achieved an average *F*_1_ score of 0.59 and average MCC of 0.29; and with RiNALMo, it achieved an average *F*_1_ score of 0.62 and average MCC of 0.35. However, the random forest classifier utilizing attention features from RNABERT showed an unsatisfactory predictive performance in all training scenarios (Figure 8B, Tables S7 and S8).

The poor performance of the random forest classifiers when trained on the smallest training batch of 20 RNA structures (RNABERT-RF with 0.06 *F*_1_ score, ERNIE-RNA-RF with 0.27 *F*_1_ score, RNA-FM-RF with 0.19 *F*_1_ score, RiNALMo-RF with 0.23 *F*_1_ score), suggests that nucleic acid LMs have not uncovered significant structural insights during pretraining and therefore lack effective few-shot learning capabilities. In contrast, the improved performance observed when the ML classifiers were trained on the largest training batch reflects the fundamental principle of ML; the more data, the better the model performance (ERNIE-RNA-RF with 0.32 *F*_1_ score, RNA-FM-RF with 0.29 *F*_1_ score, RiNALMo-RF with 0.35 *F*_1_ score when trained on the largest batch of 25715 RNA structures; RNABERT-RF was evaluated with 0.11 *F*_1_ score after training on the largest batch of 14076 RNA structures– the difference on the batch size number is due to LM input sequence length limitations). The same patterns were observed across the other examined classifiers, with exact quantification of the results provided in the SI and Figures S14 and S15. Additionally, when the images were visually inspected, the predictions were unsatisfactory (Figure 8C–F).

However, it should be noted here that visual inspection revealed a trend where RNA LMs increasingly identified true secondary contacts while progressively reducing false positives, as their architecture size grew. This finding indicates that classifiers using attention features from smaller LM architectures struggled more than those using attention from larger architectures (Figure 8C–F).

Similarly, DNABERT-3 did not manage to capture structure (up to 4.3% of attention weights aligned with the structure; Figure 9A). This result was further supported by the outcomes of the ML classifiers’ training, where all classifiers performed poorly, with again the random forest having the best performance when trained on 20 structures (0.51 average *F*_1_ score, 0.03 average MCC) or the entire training set (0.58 average *F*_1_ score, 0.27 average MCC) (Figures S12–S13 and Tables S7–S8). Visualizing the predictions using DNABERT’s attention demonstrates the failure of this LM to establish a meaningful correlation between attention and structure (Figure 9B).

**Figure 9:**
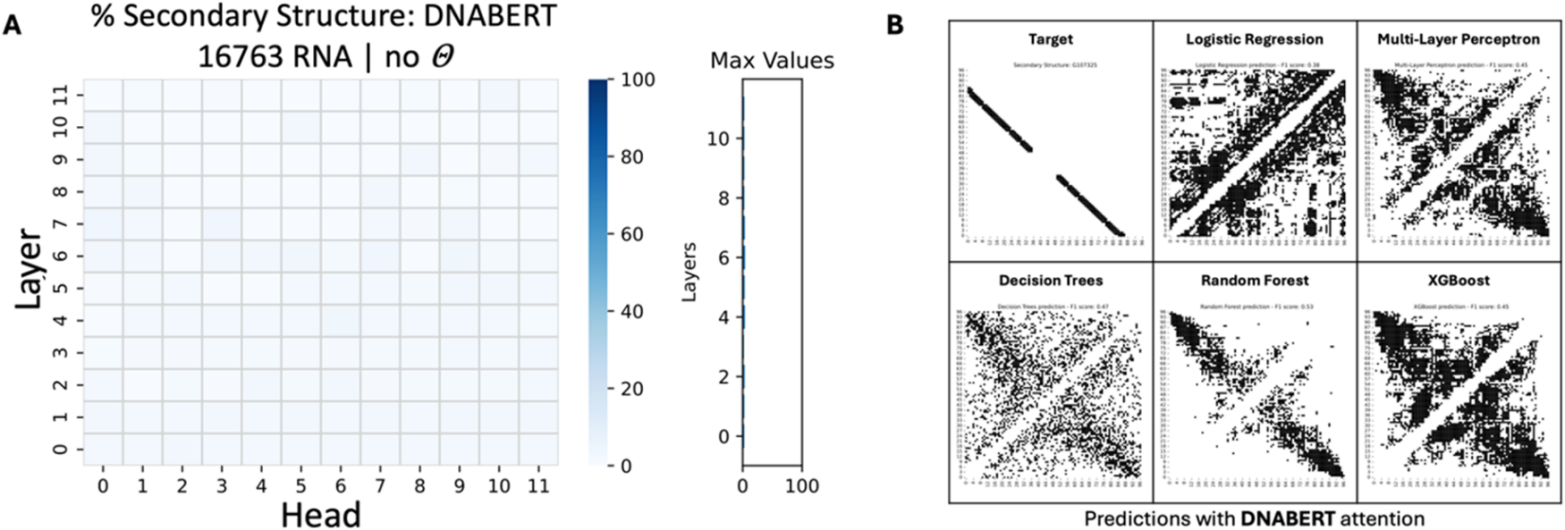
Assessment of DNABERT attention in the RNA secondary contact map prediction. (A) DNABERT failed to capture sufficient secondary structural information. The evaluation was performed over 16,763 secondary structures, which suggests that the attention mechanism of this LM did not effectively capture RNA secondary structure. (B) After visually assessing secondary contact map predictions for each classifier model (trained on the full data set), the random forest demonstrated the best performance by reducing most of the false positives (noise). However, it still fell short of providing a refined structure. (Example RNA molecule: “tdbD00010032”, source: RNAStralign^48^).

Finally, the trained CNN classifiers, which process attention maps as images to predict contact maps with a single prediction rather than separately classifying each token pair sample of the map, also yielded unsatisfactory results. The CNNs achieved low *F*_1_ scores of around 0.50 when using attention maps from either the largest or smaller nucleic acid LMs (0.54 with RiNALMo, 0.51 with RNA-FM, 0.51 with ERNIE-RNA, 0.51 with RNABERT and 0.50 with DNABERT attention) (Figure 10 and Table S9). This result further suggests that the attention mechanism of the examined nucleic acid LMs failed to capture evolutionary structural information.

**Figure 10:**
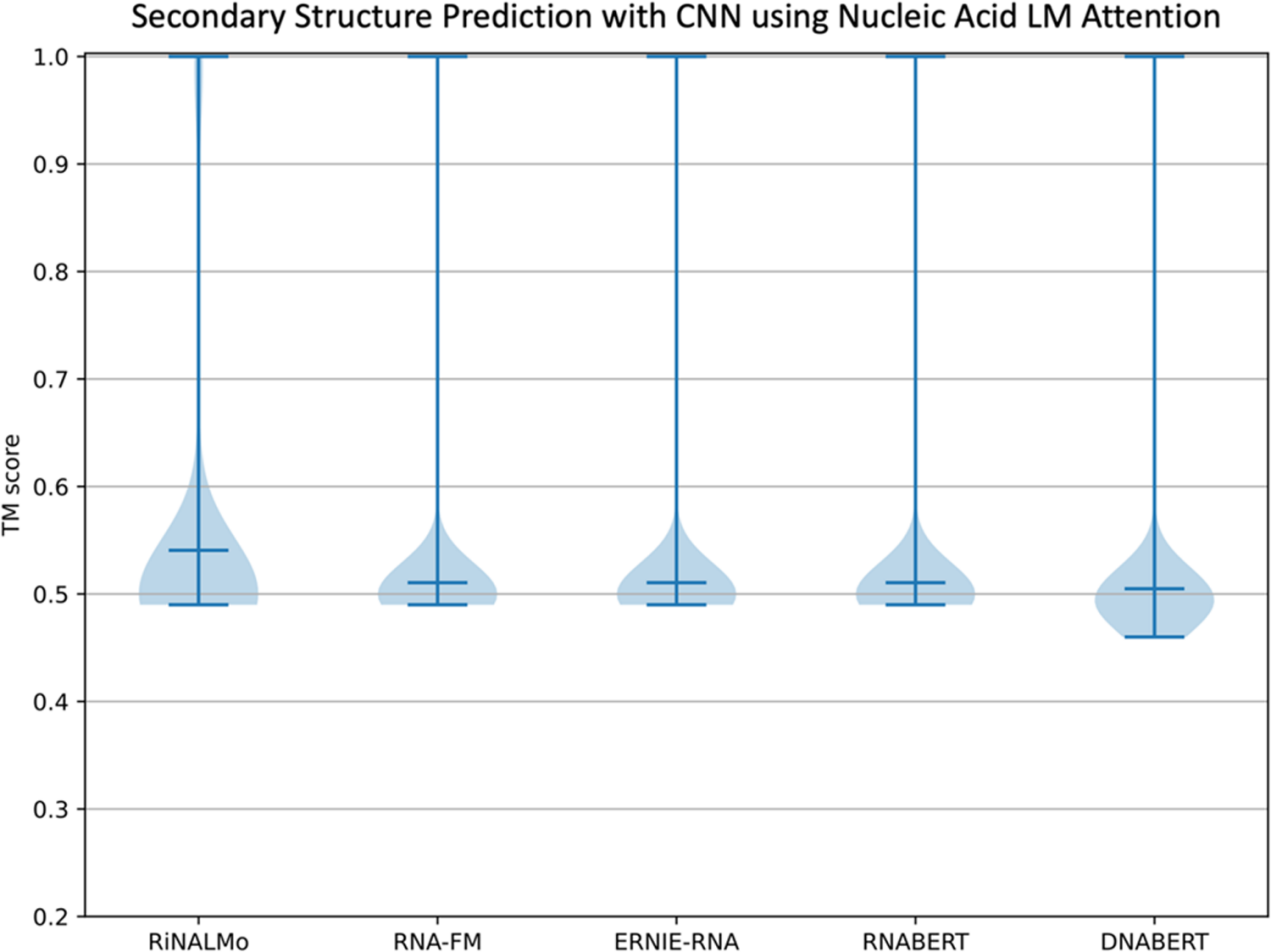
CNN classifiers designed to predict secondary structure contact maps performed unsatisfactorily on the test set when using attention maps from any of the nucleic acid LMs as input. This result reinforces the observation that the current nucleic acid LM attention weights do not directly indicate contact patterns between residues in the molecular structure, in contrast with the attention mechanism of pLMs.

### Comparison of the Developed Classifiers with the State-of-the-Art Models

To assess the performance of pretrained RNA LMs attention mechanisms in predicting RNA secondary and tertiary structure compared to state-of-the-art models, we selected the four best-performing ML classifiers from our developed models. Specifically, we chose the best-performing random forest classifiers: the ones using RNABERT attention features (RNABERT-RF), ERNIE-RNA attention features (ERNIE-RNA-RF), RNA-FM attention features (RNA-FM-RF), and RiNALMo attention features (RiNALMo-RF) trained on the full data set for secondary and tertiary structure prediction, respectively. Our classifier performance on predicting contact maps was compared to the contact maps extracted from trRosettaRNA,^36^ AlphaFold3,^37^ RhoFold+,^25^ DeepFoldRNA,^38^ and FarFar2/ARES^39,40^ for tertiary structure and SpotRNA,^41^ RNAFold,^42^ mxFold2,^43^ and RNA-MSM^23^ for secondary structure prediction.

Predicting the RNA tertiary structure for the extra data set of the 33 newly resolved RNA structures from the PDB, trRosettaRNA performed the best (0.85 average *F*_1_ score, 0.78 average MCC), along with RhoFold+ (0.82 average *F*_1_ score, 0.68 average MCC), which was set to use structural information from the LM alone and not from MSA, and AlphaFold3 (0.81 average *F*_1_ score, 0.70 average MCC). Next, DeepFoldRNA achieved an average *F*_1_ score = 0.77, average MCC = 0.68 MCC, even though it uses structural information from MSA to make its predictions, and finally, FarFar2/ARES had an average *F*_1_ score = 0.76 and average MCC = 0.61. Similar to our developed classifier models, these models achieved comparable performance, albeit there was a noticeable gap when comparing to the state-of-the-art models; RiNALMo-RF (0.58 average *F*_1_ score, 0.19 average MCC), RNA-FM-RF (0.59 average *F*_1_ score, 0.22 average MCC), ERNIE-RNA-RF (0.59 average *F*_1_ score, 0.24 average MCC), and RNABERT-RF (0.58 average *F*_1_ score, 0.23 average MCC) (Figure 11C, Tables S12 and S13). These performance values are close to random, meaning that the classifier predictions lack meaningful discriminatory power and perform similarly to a scenario where contacts are proposed without considering the underlying data patterns.

**Figure 11:**
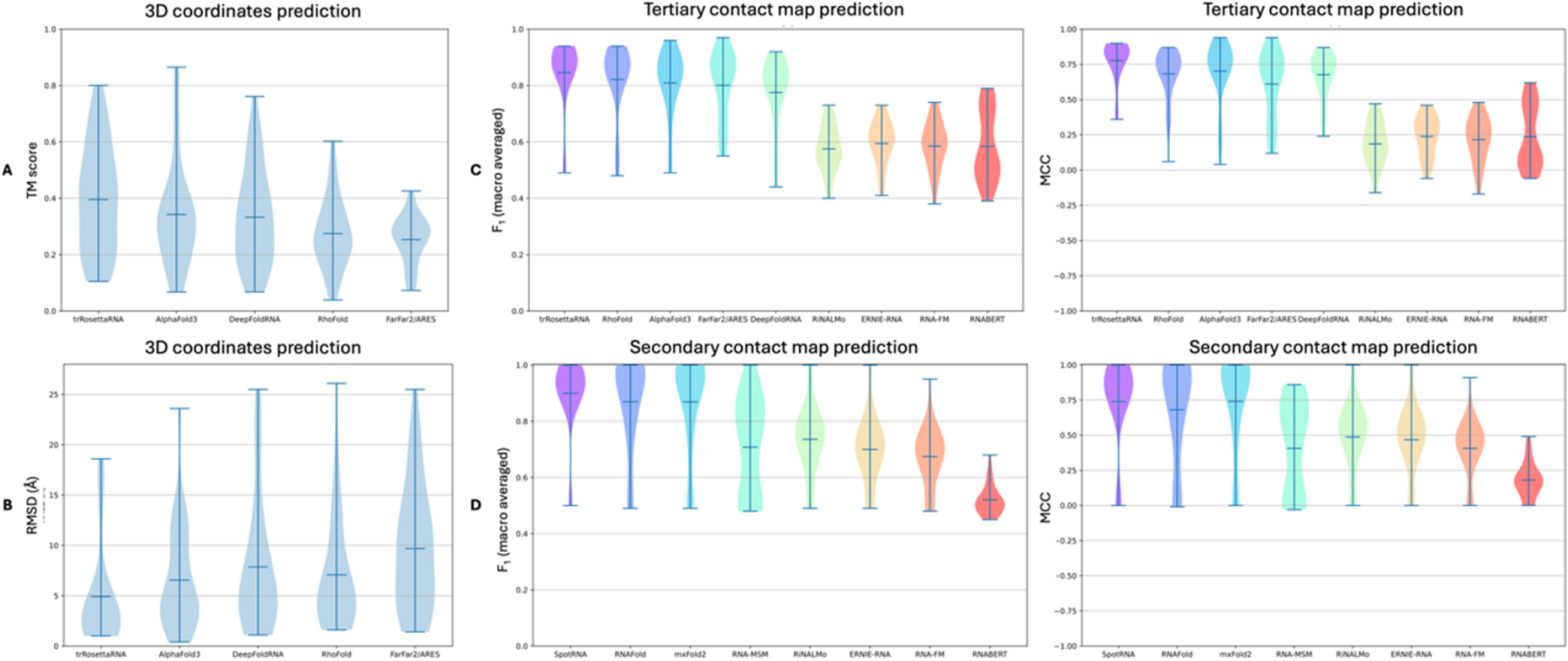
Comparison of the developed classifiers utilizing pretrained LM attention features with the state-of-the-art models for RNA structure prediction. (A, B) Violin plots display the accuracy of state-of-the-art tools (trRosettaRNA, AlphaFold3, DeepFoldRNA, RhoFold+, and FarFar2/ARES) in predicting RNA 3D structures, highlighting the superior performance of trRosettaRNA (5 Å average RMSD and average, 0.4 TM score) (see SI Section 9 for details). (C, D) Performance of developed classifiers in predicting tertiary (C) and secondary (D) contact maps, showcasing the limitations of later-developed methodologies compared with established tools.

As state-of-the-art tools can predict 3D coordinates for the RNA structure, we also evaluated their RNA structural prediction capabilities using RMSD and TM scores as additional metrics. Using these metrics allowed identifying existing limitations in predicting RNA structure. trRosettaRNA had an average RMSD = 5.0 Å and an average TM score = 0.40 and emerged as the best performer, followed closely by AlphaFold3 (6.6 Å average RMSD, 0.34 average TM score), then DeepFoldRNA (7.9 Å average RMSD, 0.33 average TM score) and RhoFold+ (7.1 Å average RMSD, 0.27 average TM score), while FarFar2/ARES (9.7 Å average RMSD, 0.25 average TM score) was the least accurate (Figure 11A,B, Tables S10 and S11). A key challenge to date lies in predicting 3D RNA folds such as G quadruplexes (PDB codes: 7MKT, 7Q48, 7PS8, 8Q4O, 8TNS), which pose a are difficult to predict due to the limited reference data available for their structures. Our developed classifiers do not predict atomic coordinates and are therefore omitted from this comparison.

In the corresponding RNA secondary structure predictions for the same data set, SpotRNA performed the best (0.90 average *F*_1_ score, 0.74 average MCC), followed by RNAFold (0.87 average *F*_1_ score, 0.68 average MCC), and mxFold2 (0.87 average *F*_1_ score, 0.75 average MCC). RNA-MSM (0.71 average *F*_1_ score, 0.41 average MCC), which uses more informative LM embeddings, achieved results similar to those of our developed RiNALMo-RF (0.74 average *F*_1_ score, 0.49 average MCC). The rest of our developed classifiers performed similarly: RNA-FM-RF (0.67 average *F*_1_ score, 0.41 average MCC), ERNIE-RNA-RF (0.70 average *F*_1_ score, 0.47 average MCC), and RNABERT-RF (0.52 average *F*_1_ score, 0.18 average MCC) (Figure 11D, Tables S14 and S15).

In conclusion, while for tertiary structure predictions, our classifiers achieved just above 50% *F*_1_ score and an MCC = 0, which is considered a random prediction, in the case of secondary structure prediction, our classifiers and specifically RiNALMo-RF and RNA-FM-RF, performed competitively well, and in some cases, even exceptionally (see Tables S14 and S15 for specific metrics). It is important to note that the improved predictions were achieved once again by the largest nucleic acid LMs, pinpointing the increased capabilities of the larger LM architecture.

## Discussion

State-of-the-art AI methodologies for predicting RNA 3D structure depend on evolutionary structural information, which can be obtained either through slow-processing MSA generation or by the utilization of experimental fragment libraries to identify conserved structural motifs and sequence patterns. However, both approaches strongly depend on the limited experimentally resolved RNA structures to generalize their knowledge and accurately predict 3D structures. This dependency becomes a limitation when predicting previously unseen RNA sequences such as synthetic and rare RNA types. We tested the performance of these methods by evaluating state-of-the-art AI methods trRosettaRNA,^36^ AlphaFold3,^37^ RhoFold+,^25^ DeepFoldRNA,^38^ and FarFar2/ARES^39,40^ on a set of 33 selected RNA to examine how they perform in predicting 3D RNA structure. Our results confirmed that these evaluated models can predict accurate structures for well-annotated RNA molecules, however, for RNA molecules with little or no available homology data, such as G quadruplexes, they yield poor structural predictions (Tables S10 and S11).

In this paper, we explore whether the attention weights of pretrained existing nucleic acid LMs can capture evolutionary 3D RNA structural information, similarly to utilizing pLM attention for protein structure prediction. First, we validate that the attention from ESM2 and ProtT5 pLMs effectively extracts amino acid contacts, which can be used to construct a protein’s 3D coordinates. Both evaluated architectures (BERT and T5) demonstrated few-shot learning capabilities, performing effectively (*F*_1_ score = 0.76–0.81) in the contact classification task. Their attention features, utilized by the ML classifier performing the task, achieved the aforementioned contact prediction performance with as few as 20 training structures and no fine-tuning.

The same methodology was reimplemented on RNA using nucleic acid LMs; however, our results show that the RNA-specific LM attention mechanisms do not capture enough structural information when utilized as features for the contact classification task for predicting RNA tertiary structure. For the less complex secondary structure, we observed a linear relationship between prediction performance, nucleic acid LM architecture size, and the depth of its pretraining. Although we found evidence of captured secondary structure-specific information inside the attention mechanisms of the largest nucleic acid LMs alignment with percentages ranging from 55 to 69.1%, as quantified in Figure 4, these models did not exhibit the same few-shot learning capabilities as pLMs. Instead, in our batch training approach, all available data (approximately 25,000) were utilized for training, yet the classifiers struggled to identify meaningful relationships between attention and secondary structure. As a result, the models failed to achieve reasonable accuracy in predicting RNA secondary structure contacts (*F*_1_ score = 0.11–0.35). Eventually, it is important to note that methods utilizing MSA to predict RNA secondary structure such as RNA-MSM, still achieved superior results compared to LMs as shown in the literature.^23^ Specifically, RNA-MSM generates attention maps that closely resemble contact maps, as demonstrated by the qualitative comparison shown in Figure 7E–G, highlighting its ability to effectively capture structural information, unlike LMs that process single RNA sequences, such as RNA-FM or RiNALMo.

At present, nucleic acid LMs face encounter considerable challenges compared to pLMs. First, nucleic acid LMs do not achieve the efficiency of pLMs, even when trained on the maximum available data, such as RNAcentral. The available 35.5M RNA sequence data from RNAcentral^5^ is approximately ∼7-fold less than the 254M protein sequence data currently available in UniProt,^71^ which directly impacts the ability of nucleic acid LMs to generalize structural patterns. However, data scarcity is not the only bottleneck. The relatively small parameter counts of nucleic acid LMs, which are ∼4-fold smaller than those of pLMs, further constrain their ability to capture intricate sequence–structure relationships. Increasing the size and parameter count of an LM could significantly enhance its ability to retrieve contextual understand of the language. Larger LMs could potentially handle more complex language patterns and offer improved few-shot learning capabilities.^72^ The advantage of larger LMs has been demonstrated in ESM-2, where increasing model parameters to 8M, 35M, 150M, 650M, 3B, and 15B learnable parameters consistently improved performance in the contact classification task without the need for additional fine-tuning.^20^ Accordingly, decoding RNA language with its limited vocabulary of just four bases is inherently more challenging than decoding the protein language of 20 amino acids. The restricted RNA vocabulary poses a challenge for smaller models to encode complex, meaningful relationships. Therefore, scaling up the size of nucleic acid LMs may enable them to capture more contextual and structural information effectively.

Another potential limitation for the use of larger RNA-specific LMs to uncover structure is the quaternary interactions influencing RNA folding. While it is the sequence that directly defines base pairing in the secondary structure, which establishes the molecule’s initial shape and guides tertiary structure formation, the tertiary structure is highly flexible in the RNA apo form. This structural flexibility declines when external ligands, such as proteins, other RNA molecules, DNA, or small molecules, bind and stabilize the 3D structure that provides RNA with its characteristic folding patterns and functionality. Therefore, even larger nucleic acid LMs may face challenges to accurately predict tertiary contacts due to these additional external factors. In this context, the recent suggested approach to predict biomolecular assemblies after processing all of their components through the same network, such as AlphaFold3 ^37^ or RoseTTAFold All-Atom,^73^ appears effective as they account for all intermolecular contributions in structure formation.

Ultimately, an RNA LM with a higher parameter count could greatly enhance the accuracy of RNA 3D structure predictions, particularly if it achieves few-shot learning capabilities and excels in contact classification along with other properties uncovered from the sequence. If its attention mechanism can capture the base interactions contributing to structure, it could enable faster and more accurate predictions compared to the current state-of-the-art models by leveraging evolutionary information learned during pretraining to address data scarcity. Moreover, while attention weights represent intermediate computations within the LM, the output embeddings, which provide a more comprehensive summary of sequence interactions, could offer greater insights for downstream tasks, such as secondary and tertiary structure prediction, functional annotation, binding affinity estimation, and mutational impact analysis similar to how pLMs decode the protein language.^74–76^ Additionally, with the development of more capable nucleic acid LMs, the potential to create approaches for predicting RNA-ligand assemblies by extracting structural features from pretrained LMs arises, offering a faster inference alternative to current MSA-based methods.

## Supplementary Material



## Data Availability

The Python code and all data sets used in this study are available at https://github.com/zoecournia/RNAstruct-LLM-Val.

## Notes

Overall methodology, available biomolecular structures collection, contact map generation, Language model selection, feature extraction and machine learning data set generation, classifier training using batches of the training molecules, predictions with convolutional neural network (Figures S1–S16 and Tables S1–S15) (PDF)
