# Supplementary material for: Predicting RNA Structure Utilizing Attention from Pretrained Language Models
Source: J Chem Inf Model. 2025 Jul 2;65(13):6483–98. doi: 10.1021/acs.jcim.4c02094 (PMC12264945; doi:10.1021/acs.jcim.4c02094)
Supplement: Supplementary file 1 [file ci4c02094_si_001.pdf]

SUPPORTING INFORMATION

Predicting RNA Structure Utilizing Attention from Pretrained Language Models

Ioannis Papazoglou[1,2,*], Alexios Chatzigoulas[1], George Tsekenis[1], Zoe Cournia[1,*]

[1]Biomedical Research Foundation, Academy of Athens, 4 Soranou Ephessiou, 11527 Athens, Greece
[2]Department of Biology, National and Kapodistrian University of Athens, 15784 Athens, Greece

1. Overall Methodology

Initially, we evaluated whether natural language processing (NLP) and self-attention can predict biomolecular structure in proteins. Furthermore, we used available language models (LMs) to train classifiers and assess whether they can predict RNA secondary and tertiary structure represented as contact maps. Finally, we compared the trained classifier models with state-of-the-art tools for RNA structure prediction. The workflow that we followed is outlined in Figure S1. Every step is described in more detail in subsequent sections.

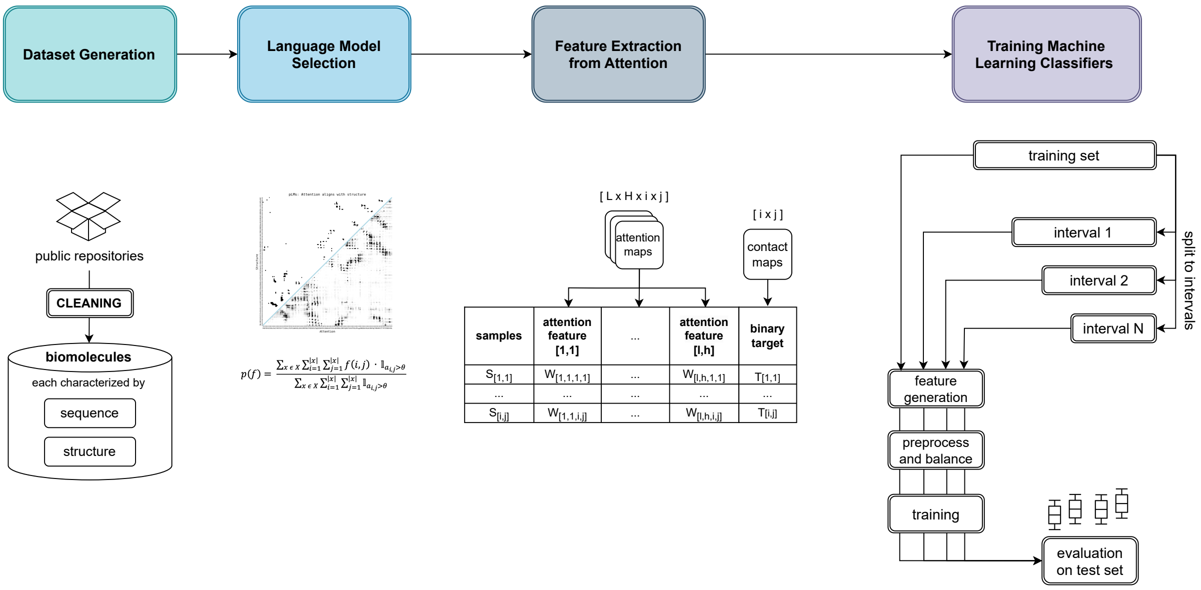

Figure S1. Workflow of our approach to predict biomolecular structure represented as contact maps: gathering accessible molecular data, choice of available pretrained LMs utilizing a statistical equation (see Equation 1 in the main text), extracting attention for feature generation, and classifier training using batches of the available training data to evaluate few-shot capabilities.

2. Collection of Available Biomolecular Structures

For testing whether natural language processing (NLP) and self-attention can be used for structure prediction in proteins, we used the trRosetta protein dataset (1) with the only modification being the exclusion of proteins larger than 1024 amino acids in sequence length due to memory limitations in GPU resources (longer sequences need more than 24 GB of VRAM). Thus, while the original trRosetta dataset consists of 15,051 proteins and their tertiary structures, we used 15,008 proteins for our experiments (Figure S2A).

To create an RNA tertiary structure dataset, we initially gathered all the available molecules with experimentally resolved structures from the Protein Data Bank (PDB) and the Nucleic Acid Knowledgebase (NDKB) (2). We curated the initial set of 929 single-stranded 3D RNA structures with a sequence length ranging from 10 to 1024 nucleotides long, which was the maximum molecule size that the selected nucleic acid LMs could process (see Section 3). In the dataset, we included apo structures and RNA bound to small-molecule ligands, but excluded RNA structures bound to large molecules due to potential holo-apo structural differences. We excluded the invalid molecules featuring unmodeled regions in their structure (implications with contact map generation) or containing modified or unusual bases in their sequence (could not be processed by nucleic acid LMs). In cases where multiple resolved models existed, we randomly selected one as the representative structure.

For the RNA secondary structure dataset generation, we merged bpRNA (3), RNAStralign (4), and ArchiveII (5). These databases did not contain experimentally determined RNA structures, but instead offered high-quality, computationally calculated secondary structures derived through energy calculations and alignments with known homologous data. The obtained dataset collectively provided an extensive pool of 143,354 molecules, from which, similarly to the RNA tertiary structure dataset, we removed molecules with modified or unusual bases and kept molecules with sequence lengths ranging from 10 to 1024 nucleotides.

Finally, we reduced the redundancy within the RNA datasets by removing similar or duplicate sequences using the CD-HIT-EST algorithm (6), thereby eliminating any highly correlated information that could potentially affect our experiments. We configured the CD-HIT-EST algorithm to cluster sequences with a minimum of 90% similarity in length and set an 80% similarity threshold after global alignment. Additionally, we instructed the algorithm to choose a random sequence from each cluster as the representative. After this last step, 425 molecules with known tertiary structures and 30,252 RNA

molecules with their secondary structures remained in the tertiary and secondary RNA structure datasets, respectively (Figure S2B and S2C).

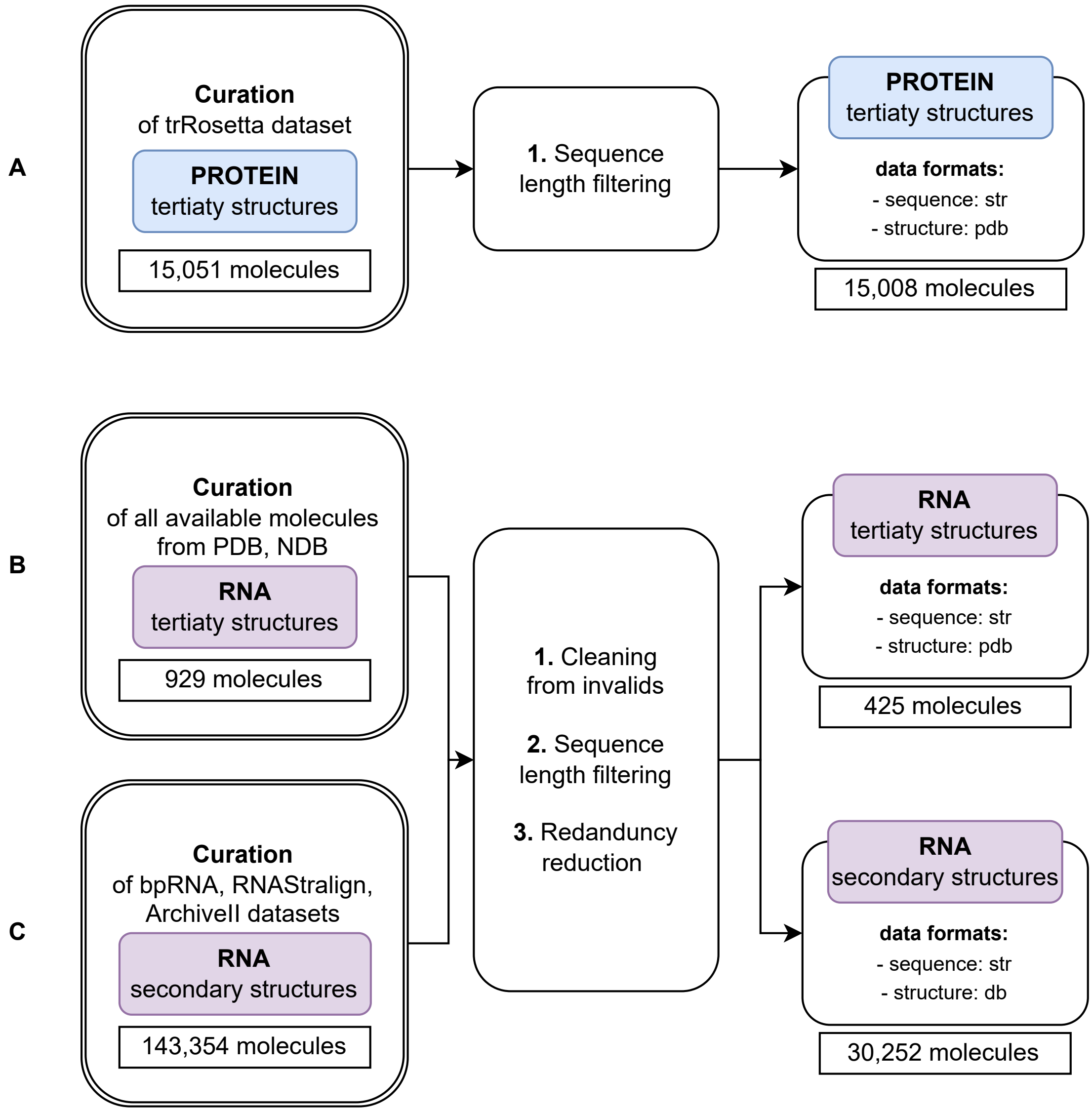

Figure S2. A. 15,008 proteins were curated from the trRosetta dataset and used for the validation of the methodology in predicting contact maps representing tertiary protein structure. **B.** 425 RNA molecules with known tertiary structure and **C.** 30,252 RNA molecules with known secondary structure were used to re-implement the methodology for RNA tertiary and secondary contact map prediction, respectively.

3. Contact Map Generation

To generate contact maps for tertiary structure data saved in the atomic coordinate file format of the PDB, both for proteins and RNA, we developed a Python script that inputs atomic coordinates utilizing the Biopython (7) library and computes the distances between all heavy atoms between every residue pair. Then, it selects the shortest calculated distance as the distance between all residue heavy atoms, thus creating a distance map. Finally, by using a cutoff distance it creates the final binary contact maps. We evaluated three distance cutoffs (8 Å, 9.5 Å, and 12 Å) for generating the contact maps. These cutoffs were evaluated for their index in attention weights indicating structure with the method explained in the main text (Methods; Language Model Selection - Attention Mechanism Assessment). Our findings indicated consistent signal patterns across the attention heads for all tested cutoffs (Figure S3, S4). Notably, the 12 Å threshold exhibited a higher percentage of structural attention alignment compared to the 8 Å threshold. We ultimately chose 9.5 Å as it strikes a balance, avoiding both overly strict and excessively loose representations in the contact map.

Also, as suggested by Meta AI, we ignored contacts between nearby amino acids that are in the range of 6 positions (i ± 6) and nucleotides in the range of 4 positions (i ± 4), by setting their binary value as 0 in the generated contact maps. We ensured that this change did not result in information loss, as these contacts refer to the primary structure, which is already known by the sequence data. Furthermore, these contacts were found to be beneficial for downstream experiments (Figure S3).

Similarly, to generate contact maps that depict secondary structure, we wrote another Python script to translate the dot-bracket notation in binary contact maps. We paired open and closed parentheses or brackets within the dot-bracket notation input and then mapped their positions to binary values in the resulting contact map (Figure S5). The generated maps accurately represented the secondary structural relationships within the RNA molecule.

For calculations utilizing DNABERT, contacts had to be represented in the context of 3-mers as the selected version of the LM operates on 3-mers rather than individual 1-mers (see Section 4). This was a novel task for us as traditional contact maps indicate relationships between single residues (1-mers). For the contact maps representing tertiary structure, we followed the same process as before, with the difference that we now measured all heavy atom distances between every occurring 3-mer instead of a single residue. To generate contact maps representing secondary structure in a 3-mer fashion, we designed a Python script that employs a scanning process using a kernel of size 3x3. This kernel iterates over the binary map, and for each position, it checks if the surrounding 3x3 window contains at least one “1” value. If so, it assigns

the value of the corresponding 3-mer position in the generated map to “1”; otherwise, it assigns a “0” value (Figure S6).

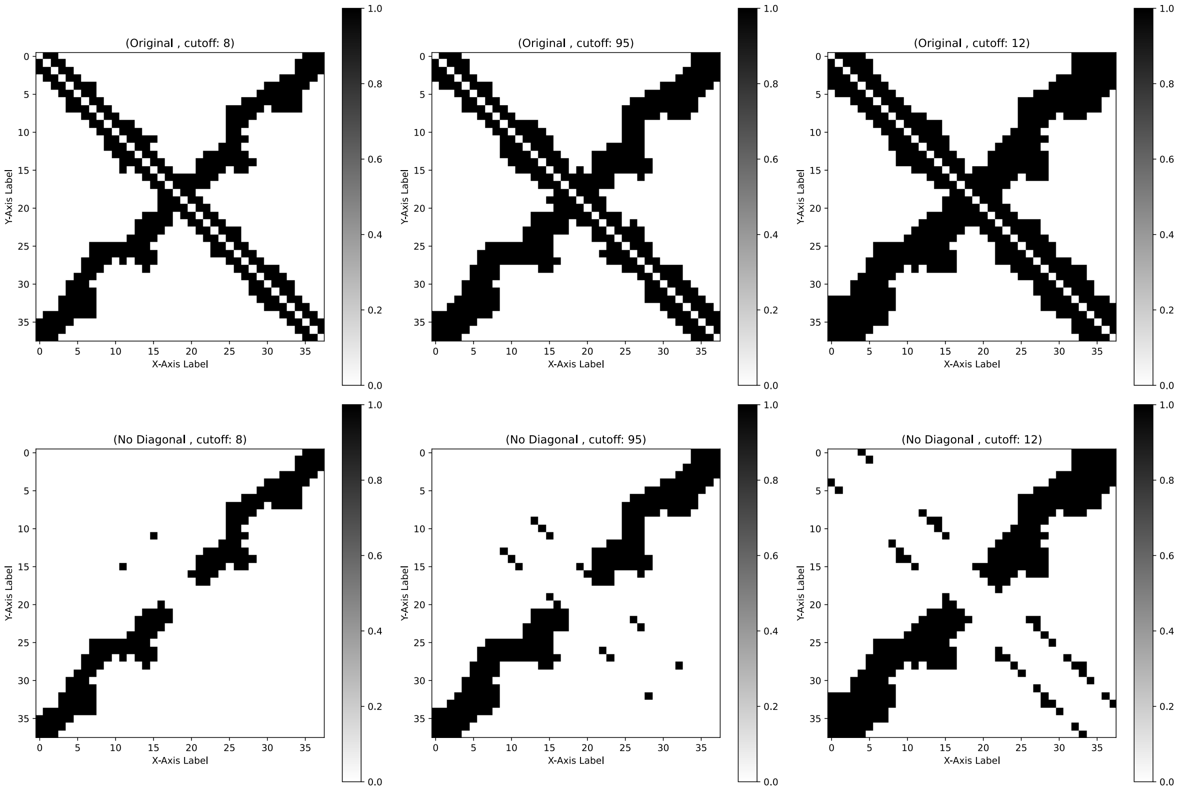

Figure S3. Generated contact maps using different distance cutoffs (8 Å, 9.5 Å, 12 Å), including the main diagonal representing local contacts if the primary structure (top) or ignoring them (bottom).

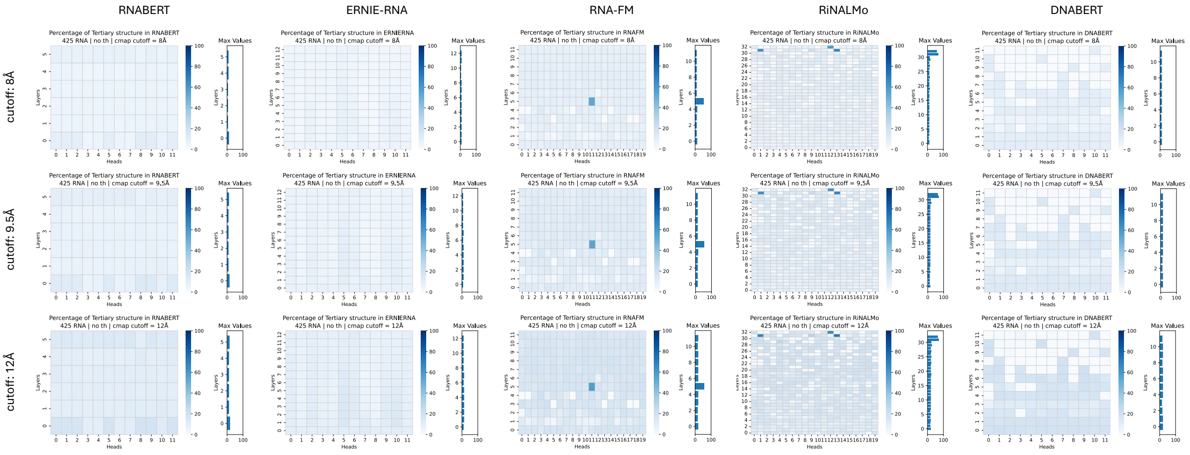

Figure S4. Attention heads of 425 tertiary RNA structures using different cutoffs (8 Å, 9.5 Å, 12 Å) using the five nucleic acid LMs. As anticipated, certain heads consistently showed higher proportions of attention weights indicating structure. With minor differences, the cutoff of 9.5 Å emerged as the ideal cutoff because it strikes a balance, avoiding the over-generalization seen at 8 Å and over-specialization seen at 12 Å in the resulting contact maps.

Base Sequence: A C C U G G G U
Secondary Structure: . ((. .)) .

	A	C	C	U	G	G	G	U
**A**	0	0	0	0	0	0	0	0
**C**	0	0	0	0	0	0	1	0
**C**	0	0	0	0	0	1	0	0
**U**	0	0	0	0	0	0	0	0
**G**	0	0	0	0	0	0	0	0
**G**	0	0	1	0	0	0	0	0
**G**	0	1	0	0	0	0	0	0
**U**	0	0	0	0	0	0	0	0

Figure S5. Visual representation of the conversion process of secondary structure data from dot-bracket notation to a binary contact map: base pairs indicated with parenthesis are depicted with a “1” value in the resulting contact map.

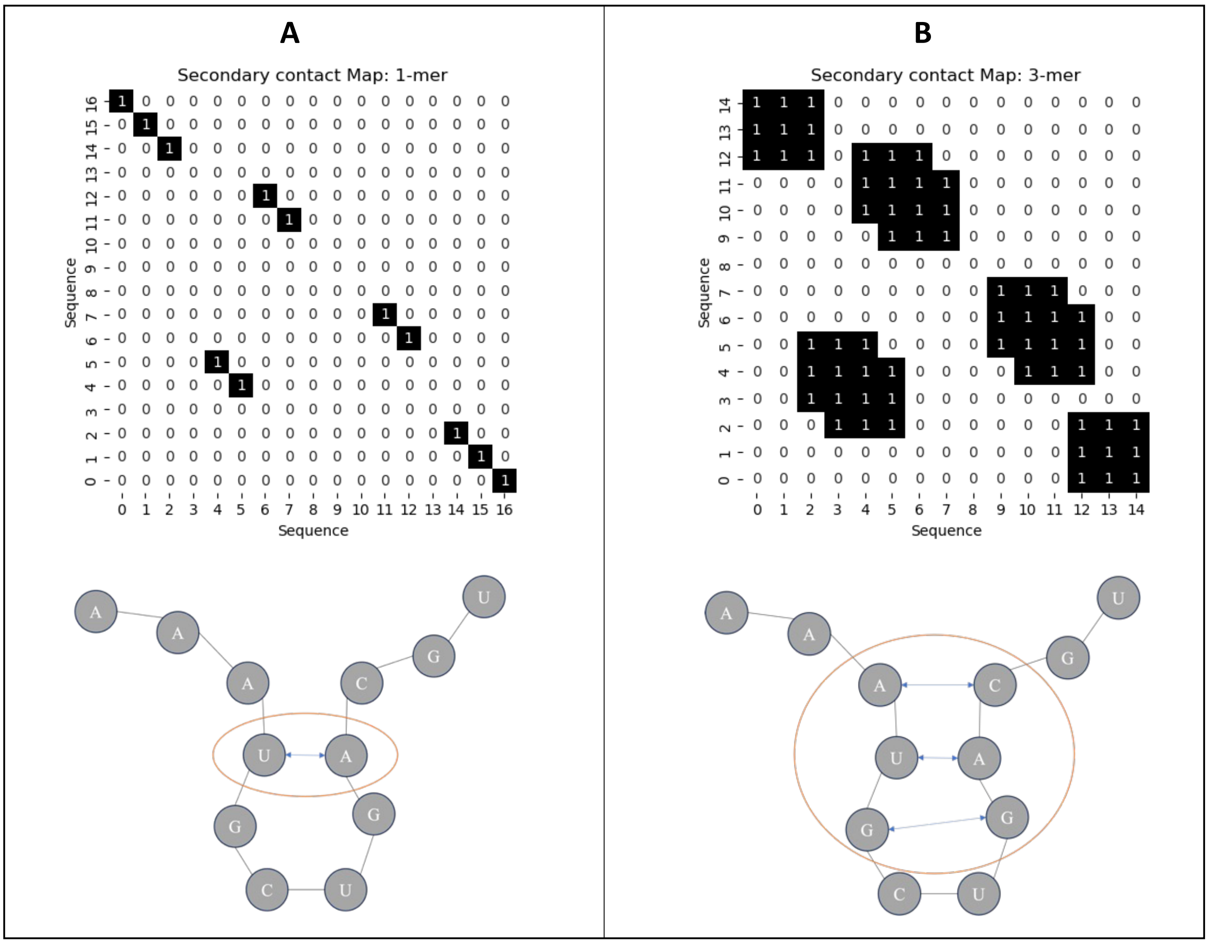

Figure S6. If two residues are at a distance with a certain cutoff, then their immediate neighboring residues will also be close to each other. This is the biological assumption on which the kernel methodology and 3-mer contact map generation methodology were based; **A**. Contact maps for 1-mer tokenization **B**. Contact maps for 3-mer tokenization.

4. Language Model Selection

For the protein dataset, we utilized the evolutionary Scale Model (ESM-2) (8) developed by Meta-AI with the BERT architecture and 1-mer tokenization, which was pretrained on a total of 60 million protein sequences (version of 3 billion parameters). Additionally, we employed two versions of the ProtTrans (Prot-T5) (9) model, developed by RostLab, that utilize the T5 architecture and 1-mer tokenization. One of them was pretrained on 45 million sequences from uniref50 (10) (uniref50-XL version) and the other on 2.1 billion from BFD (11, 12) (bfd-XL version). We used these pLMs through the Hugging Face library with the option to extract the attention weights (*output_attentions*=True).

For the RNA datasets, we used RNABERT (13), RNA Foundation Model (RNA-FM) (14), ERNIE-RNA (15), RiboNucleic Acid Language Model (RiNALMo) (16) and DNABERT-3 (17). We employed the LMs after

reloading the weights offered by their developers after their pretraining (18). For RNABERT, we modified the source code, because it was designed to output only the final embeddings. Thus, we modified its multihead self-attention module to output the attention maps as well. For the RNA-FM, ERNIE-RNA and RiNALMo implementations, we followed the guidelines provided in their respective GitHub repositories. For each model, the required environments were set up using the corresponding dependencies and configurations. Finally, we utilized the provided inference scripts for the extraction of attention weights. For DNABERT-3, we utilized it to process RNA sequences subject to the assumption that DNA and RNA languages share significant similarities through their co-evolution. Consequently, we processed each RNA sequence by converting them into DNA sequences (U to T conversion). DNABERT-3 was implemented by directly loading the model and its associated tokenizer from the Hugging Face model hub, with configuration to enable the extraction of attention weights (*output_attentions*=True). Additionally, DNABERT-3 tokenizes sequences into 3-mers, which differs from our methodology's requirement of 1-mers. To align with DNABERT, we employed a scanning process using a 3x3 kernel to convert our target contact maps into 3-mers. When the kernel identified a positive value (indicating the presence of a contact), it was marked as '1'; otherwise, it was marked as '0' in the newly generated 3-mer contact map. The conversion method is described in Section 3.

All relevant scripts or configurations are available in the supplementary materials or GitHub repository for reproducibility (https://github.com/zoecournia/RNAstruct-LLM-Val).

5. Feature Extraction and Machine Learning Dataset Generation

All attention maps, which were obtained after processing molecules through LMs, were preprocessed following Meta AI's recommendations with symmetrization (8) and Average Product Correction (APC) (18). Symmetrization involves assigning a common value to the corresponding elements on both sides of the matrix (map) diagonal and is described by equation 1.

$$x_{sym}[i,j] = x[i,j] + x[j,i] \quad (1)$$

The new value is the average of the two corresponding element values. Symmetrization transforms the attention maps into symmetrical representations, similar to the symmetrical nature of contact maps. APC removes the general trend (average progression) of values from each element. By subtracting the average progression from each element value, the focus changes to specific differences rather than the general pattern, enabling the classifiers to capture more meaningful information.

For dataset generation, we treated each token pair as an individual sample. The features for each sample consisted of the preprocessed pairwise attention weights assigned to it by the respective LM, while the target variable was the corresponding binary value from the contact map (Figure S7). However, the generated datasets suffered from significant class imbalance, with approximately 98% of the token pair samples being in class “0” (non-contacts). To address this issue, we employed the RandomUnderSampler class from the imbalanced-learn library (19), which randomly keeps instances from the majority class, resulting in balanced datasets, where both classes are represented equally or nearly equally to avoid bias towards the majority sample class.

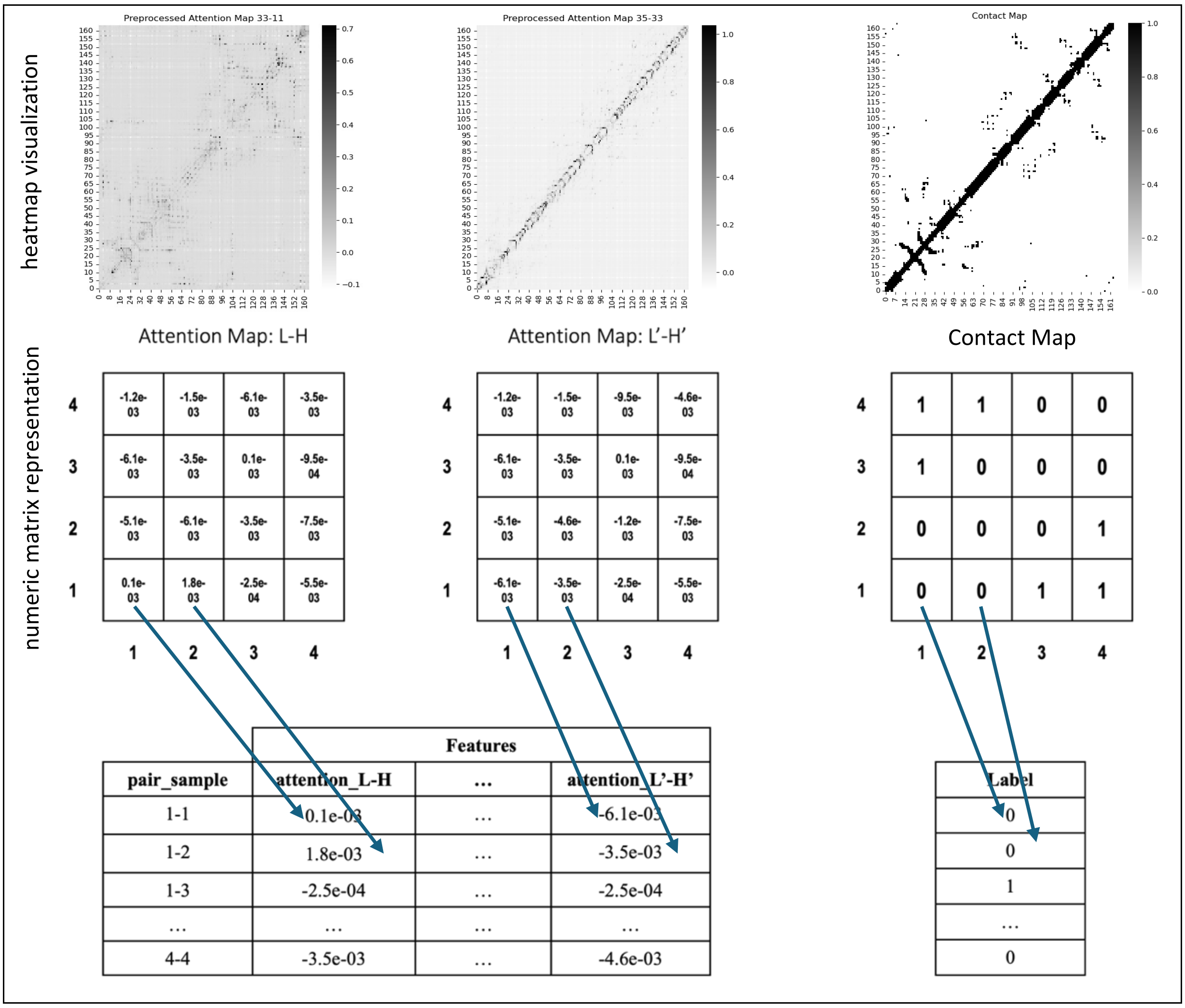

Figure S7. Feature generation from attention; every token (base) pair is considered a single sample, while the corresponding attention weights assigned to it by each LM are utilized as features. The binary value of the token pair from the contact map serves as the target.

6. Classifier Training Using Batches of the Training Molecules

For the protein structure predictions, as Meta AI suggests in their paper (8), we only trained a logistic regression classifier in a dataset generated utilizing 20 randomly selected proteins, following the dataset generation methodology described in section 1. We then used the rest 14,988 molecules as test instances and evaluated the predictions with the F_1 score (macro averaged) and Matthew's correlation coefficient (MCC) metrics.

For both tertiary and secondary RNA structure predictions, we trained multiple classifiers: logistic regression (20), decision trees (21), random forest (22), and a multilayer perceptron (23) from the scikit-learn library (24), and the XGBoost (25) classifier (Table S1). We performed training in batches of the initial training set molecules, ranging between 20 molecules to all instances, in order to examine how the performance of the models changes or converges as we vary the amount of training data used.

In particular, the 425 RNA molecules with tertiary structure were separated in 85% as the training subset (362 molecules) and 15% as the test subset (63 molecules). The training set was then further partitioned into batches of 20 (~5%), 60 (~15%), 140 (~40%), and 240 (~60%) molecules each. For the 30,252 RNA molecules with a secondary structure that could be processed by the nucleic acid LMs, we divided them in a similar fashion: 85% were used as the training set (25,715 molecules) and 15% as the test set (4,537 molecules). Again, the training set was subdivided into batches of 20 (~0.01%), 2,500 (~10%), 5,000 (~20%), and 8,000 (~30%) molecules each. It is important to note here that ERNIE-RNA developers recommend excluding sequences longer than 512 nucleotides, as processing them in chunks may omit crucial structural and functional information. However, because the model supports this chunking approach for longer sequences, such as RNA-FM and RiNALMo, we chose to include sequences up to 1024 nucleotides long. For RNABERT and DNABERT, which accept molecules up to 412 and 512 sequence length, respectively, we utilized only the shorter molecules of the initial dataset, after splitting it again into a training set, the same number of training batches and test set (RNABERT: 16,560 total; 14,076 training; 2,484 test. DNABERT: 16,763 total; 14,102 training; 2,661 test). While generating the batches, we also confirmed that their distributions encompassed a representative number of molecules from every sequence range. This step ensured better training conditions by maintaining diversity across the batch. The splitting scheme is summarized in Figure S8.

We prepared the training datasets following the dataset generation methodology described in section 1 for every batch in addition to the entire training set and then trained the machine learning classifiers.

Eventually, we assessed their predictions on the test molecules using the F_1 score (macro averaged) and the MCC metrics.

The F_1 score serves as the harmonic mean of precision and recall (Equations 2-4), providing a balanced assessment of the model's performance:

$$F_1\ score = \frac{2 * precision * recall}{precision + recall} \quad (2)$$

$$precision = \frac{TP}{TP + FP} \quad (3)$$

$$recall = \frac{TP}{TP + FN} \quad (4)$$

The MCC score (Equation 5) considers all four quadrants of the confusion matrix:

$$MCC = \frac{TP * TN - FP * FN}{\sqrt{(TP + FP)(TP + FN)(TN + FP)(TN + FN)}} \quad (5)$$

MCC ranges from -1 to +1, where +1 indicates a perfect prediction, 0 indicates random prediction, and -1 indicates total disagreement between prediction and observation. MCC achieves high values only when the classifier model performs well across both positive and negative instances.

An individual F_1 and MCC score was measured for each of the predicted contact maps. Eventually, the mathematical average of all the occurring F_1 and MCC scores referring to individual molecules of the test set was used to evaluate the overall performance of each classifier.

Table S1. Selected machine learning classifiers; hyperparameters used for the training (most are defaults and no hyperparameter optimization was performed).

**Logistic Regression**	
Regularization penalty	l2
Regularization strength (C)	1.0
Solver	lbfgs
Optimization tolerance	1e-4
Class weights	None
Maximum iterations	1000
**Decision Tree Classifier**	
Split criterion function	gini
Split strategy	'best'
Maximum depth of tree	No limit
Minimum samples to split	2
Minimum samples per leaf	1
Maximum features used for split	All features
Maximum leaves	No limit
Class weights	None
**Random Forest Classifier**	
Number of estimators (trees)	100
Criterion	gini
Maximum depth of tree	No limit
Minimum samples to split	2
Minimum samples per leaf	1
Maximum features used for split	Sqrt(features)
Maximum leaves	No limit
Class weights	None
**Multilayer Perceptron**	
Hidden layer size	100
Activation function	ReLU
Solver	Adam
Strength of L2 regularization (alpha)	0.0001
Batch size	200
Learning rate	0.001
Learning rate schedule	constant
Maximum iterations	200
Shuffle samples per iteration	True
Optimization tolerance	1e-4
Early stopping	False
**XGBoost classifier**	
Booster (model)	gbtree (tree-based)
Loss function (objective)	Squared error
Learning rate (eta)	0.3
Maximum depth of tree	6
Maximum leaves	No limit
L2 regularization (lambda)	1
L1 regularization (alpha)	0
Scaling (for imbalanced sets)	0

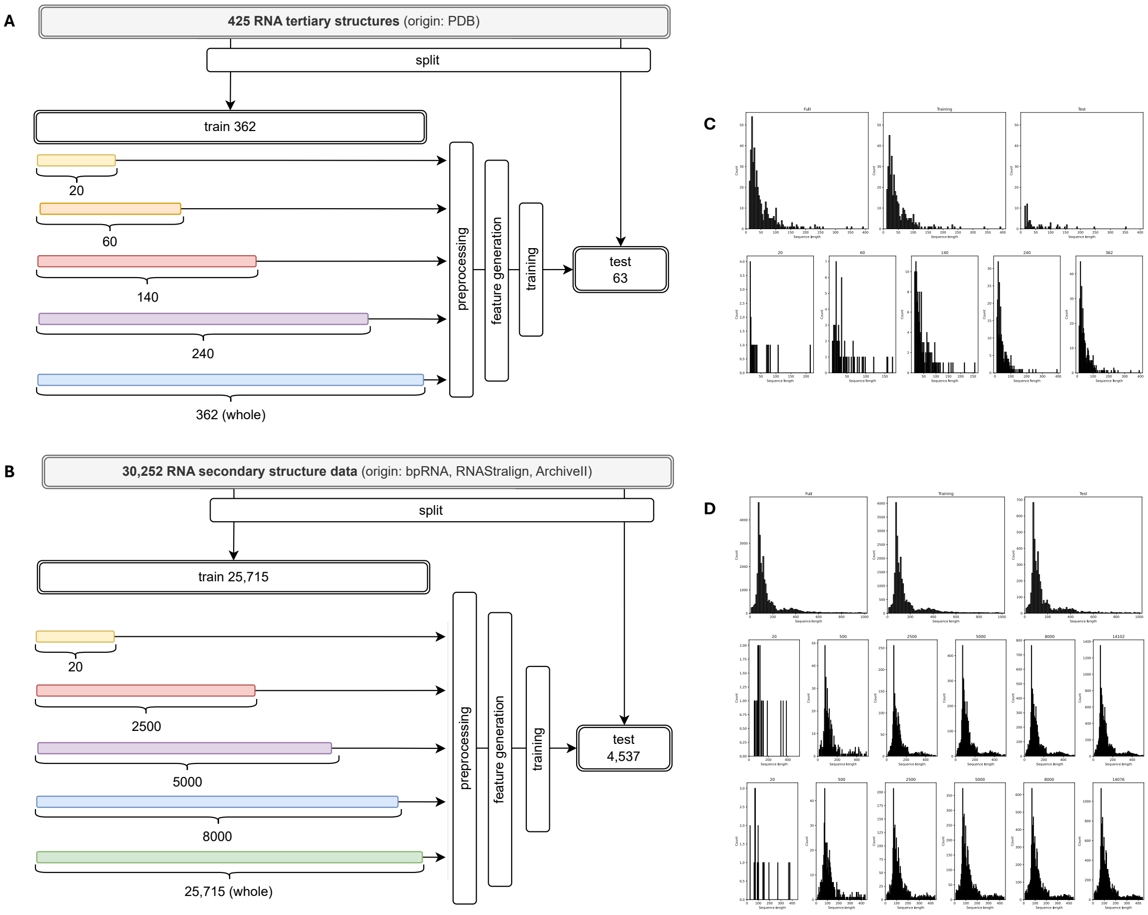

Figure S8. The training in batches approach that we employed for RNA. **A**. tertiary and **B**. secondary structure predictions. **C**, **D**. The distributions display the representative number of molecules from every sequence range that was selected for each batch training.

7. Predictions with Convolutional Neural Network (CNN)

Additionally, for the vast amount of RNA secondary structure data, we also trained a Convolutional Neural Network (CNN) (26) classifier with PyTorch (27) that could process the attention maps extracted from the corresponding LMs as images. In these images, each pixel in position (i,j) corresponded to the attention weights that the LM assigned to the respective token pair (i,j). The sum of attention maps extracted from each head of the architecture was added to the input image as multiple channels.

The architecture that we developed comprised a simple artificial neural network (fully connected layer) on top of two convolutional and pooling layers that are used to automatically generate features for the classification, by applying a series of filters across the input image to create feature maps. Pooling layers are then used to downsample these feature maps, reducing their dimensions and consequently, the computational complexity of the network. Finally, the fully connected layer contains a neural network that inputs the highest-level feature maps extracted by the convolutions and makes the final predictions in a 1D binary vector, which is lastly fed into a SoftMax function. When transposed to the initial dimensions of the input data, this vector reveals the predicted contact maps of the molecule (Figure S9A, Table S2).

The images used for CNN evaluation were preprocessed with symmetrization and APC and also padded with “zero” pixels, which was implemented by appending rows and columns of 0 values to extend the images in both dimensions in order to reach the universally set maximum dimensions of 1024 x 1024 and 660 channels for RiNALMo, 240 channels for RNA-FM, 156 channels for ERNIE-RNA, 144 channels for DNABERT or 72 channels for RNABERT attention images (Figure S9B). The CNN was trained using the binary cross entropy (BCE) loss function from PyTorch (27) in the same training datasets as with the other machine learning classifiers. Through training and validation, we used masking to crop the padded pixels (value = 0) to pass the original images to the loss function, and we handled the imbalance by using class weights that we provided in the ss function. These class weights were calculated for each molecule as suggested by scikit-learn (24) (Equation 6):

$$class\ weights = \frac{n_{samples}}{n_{classes} * y} \quad (6)$$

where $n_{samples}$ is the number of pairs inside the molecule, $n_{classes}$ is the number of classes, and y is the sample size of each class. The calculated class weights were augmented by an additional coefficient to further reward the contact prediction, addressing the challenge posed by the highly imbalanced target.

We determined that setting this coefficient to 10 yielded optimal results through trial and error. The predicted contact maps were once again evaluated using the F_1 (macro averaged) and MCC metrics.

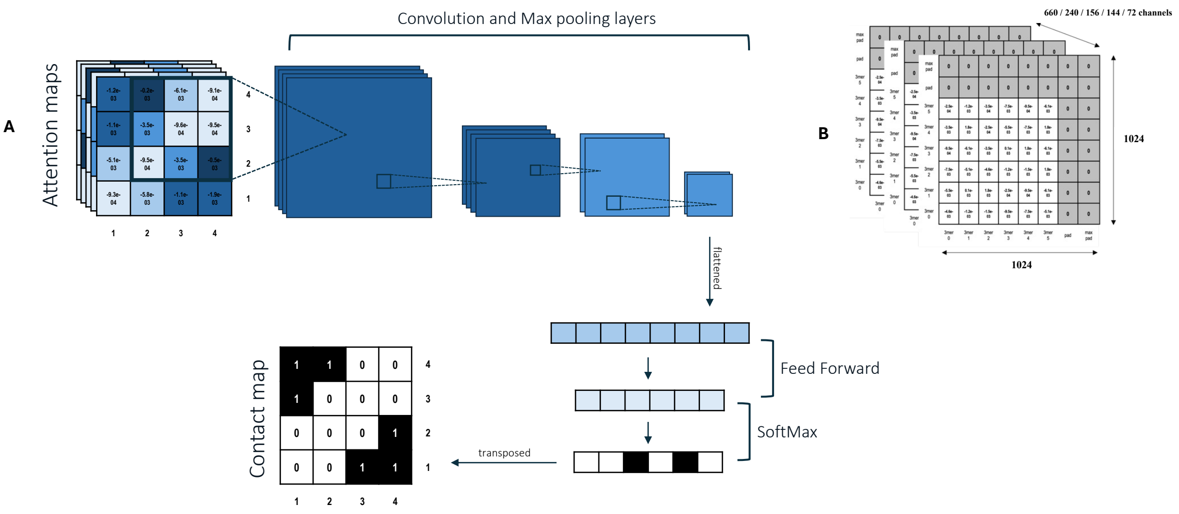

Figure S9. The CNN architecture designed for contact classification; **A.** The CNN learns spatial features by scanning the input attention maps through a series of two convolutions and pooling layers, **B.** Attention maps input; padded images of 1024x1024 pixels and 660 / 240 / 156 / 144 / 72 channels extracted from RiNALMo / RNA-FM / ERNIE-RNA / DNABERT / RNABERT, respectively.

Table S2. CNN specifications; contact classification from LM attention.

Layer	Output Dimensions	Purpose / Specifications
Input	(1, LxH, 1024, 1024)	Represents attention maps as images.
1st Conv2d	(1, 64, 1024, 1024)	Extracts features from input maps (3x3 kernel, ReLU).
1st MaxPool2d	(1, 64, 512, 512)	Reduce spatial dimensions by half.
2nd Conv2d	(1, 32, 512, 512)	Further extracts features (2x2 kernel, ReLU).
2nd MaxPool2d	(1, 32, 256, 256)	Further reduces dimensions by another half.
Flatten	(1, 2097152)	Flattens the feature maps into one single-dimensional feature vector.
1st Linear	(1, 128)	First fully connected layer to project features into a new space (ReLU).
2nd Linear	(1, 1048576)	Final fully connected layer to output logits reshaped to a square grid.
SoftMax	(1, 1048576)	Converts logits to probabilities for each class/output.
Transpose	(1024, 1024)	Returns the flattened contact map into a matrix.
Padding exclusion	(length, length)	Recovers only the binary values indicating the RNA contact map.

8. Comparison with State-of-the-art RNA Structure Prediction Tools

For our benchmark, we curated a set of 33 RNA molecules (Table S3) from the PDB, to which we performed tertiary structure predictions using trRosettaRNA (28), AlphaFold3 (29), DeepFoldRNA (30), and FarFar2/ARES (31, 32), and for secondary structure predictions, we utilized SpotRNA (33), RNAFold (34), mxFold2 (35), and RNA-MSM (36).

Initially, we evaluated the 3D predictions individually, assessing their accuracy using metrics such as Root Mean Square Deviation (RMSD) (Equation 7) and Template Modeling (TM) score (Equation 8) to evaluate the resulting structures. The RMSD score quantifies the overall difference or deviation between the predicted and a reference structure. A lower RMSD value indicates a better match between the two structures. It particularly calculates the average distance between corresponding atoms of the two structures after they are optimally superimposed (37):

$$RMSD = \sqrt{\frac{\sum_{i=1}^{N}(x_i - \hat{x}_i)^2}{N}} \quad (7)$$

where N is the number of all the included in the calculation atoms, x_i is the actual atomic position and $\hat{x}_i$ is the predicted atomic position. Specifically for our estimations, we calculated the distances between the sugar backbone and phosphate atoms. Additionally, the TM score quantifies the structural similarity by measuring the degree of overlap and alignment between corresponding residues in the predicted and experimental structures:

$$TM\ score = \max\left[\frac{1}{L_{target}}\sum_{i}^{L_{common}}\frac{1}{1+\left(\frac{d_i}{d_0\left(L_{target}\right)}\right)^2}\right] \quad (8)$$

where d_i is the distance between the i-th pair of residues in the predicted and experimental structures after superimposition.

To compare our developed classifier models (nucleic acid LM attention and random forest classifier trained on the full dataset) with the selected state-of-the-art tools, we converted the predicted 3D coordinates or dot-bracket notation strings into binary contact maps. This conversion was facilitated using the scripts

previously described (sections 2 and 3). Subsequently, we assessed and compared their performance using the F_1 score and MCC metrics.

Table S3. Properties of the 33 RNA benchmarking molecules. With the “+” sign, G quadruplexes are indicated, as the most difficult indices to predict.

**PDB code**	**Date** (DD/MM/YY)	**Origin**	**Length**	**Similar molecules** in PDB
7KUB (38)	1/6/22	natural	60	0
7KUC (38)	1/6/22	natural	16	0
7KUD (38)	1/6/22	natural	13	0
7MKT+ (39)	2/11/22	synthetic	23	0
7PS8+ (40)	5/4/23	natural	23	0
7Q48+ (40)	16/11/22	natural	22	0
7RWR (41)	20/7/22	synthetic	38	0
7SHX (42)	19/10/22	natural	94	0
7UCR (43)	20/7/22	natural	27	42
7UMC (44)	6/7/22	natural	70	3
7UMD (44)	6/7/22	natural	40	0
7UME (44)	6/7/22	natural	28	0
7UR5 (45)	10/8/22	natural	90	4
7UZ0 (46)	8/6/22	natural	87	2
7V06 (47)	29/6/22	natural	43	3
7WIA (48)	18/1/23	natural	50	5
8BWT (49)	30/8/23	natural	26	0
8CLR (50)	19/7/23	synthetic	14	0
8CQ1 (51)	20/9/23	natural	44	0
8D28 (52)	30/11/22	synthetic	33	14
8FB3 (53)	22/2/23	natural	34	5
8FCS (54)	22/2/23	natural	71	0
8FZA (55)	30/8/23	natural	30	0
8HB8 (56)	22/3/23	natural	55	5
8I44 (57)	29/3/23	natural	19	3

8ITS (58)	27/3/24	natural	46	0
8JHP (59)	29/5/24	natural	27	0
8Q4O+(60)	6/3/24	natural	23	0
8SCF (61)	12/7/23	natural	30	0
8SCH (61)	12/7/23	natural	68	0
8THV (62)	2/8/23	natural	29	1
8TNS+ (63)	15/11/23	natural	24	0
8UPT (64)	10/1/24	natural	71	0

9. Supplementary result figures and tables

$$p(f) = \frac{\sum_{x\,\epsilon\,X} \sum_{i=1}^{|x|} \sum_{j=1}^{|x|} f(i,j) \cdot \mathbb{1}_{a_{i,j}>\theta}}{\sum_{x\,\epsilon\,X} \sum_{i=1}^{|x|} \sum_{j=1}^{|x|} \mathbb{1}_{a_{i,j}>\theta}} \qquad (9)$$

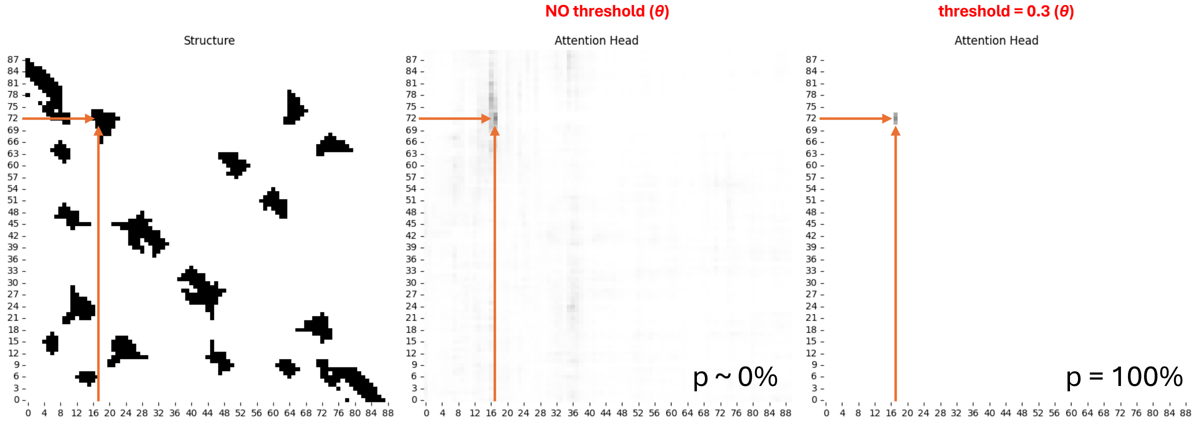

Figure S10. The provided function measures the percentage of attention weights from an attention map that aligns with a contact map (attention – structure alignment). When a threshold (e.g., 0.3) is applied, weaker attention weights below the threshold are excluded from both the numerator and the denominator, and only the strongest remaining weights are evaluated. If these few weights happen to align with the contact map, the function calculates a perfect alignment score (100%). However, this does not truly reflect the actual alignment across the full attention map, which is close to zero. Instead, it represents a localized alignment of the strongest weights. This phenomenon is only seen when evaluating the attention of nucleic acid LMs and is likely related to how they learn and distribute their attention weights. To avoid this unfair bias and ensure a comprehensive evaluation, we opted not to use a threshold when evaluating the nucleic acid LMs with Equation 9.

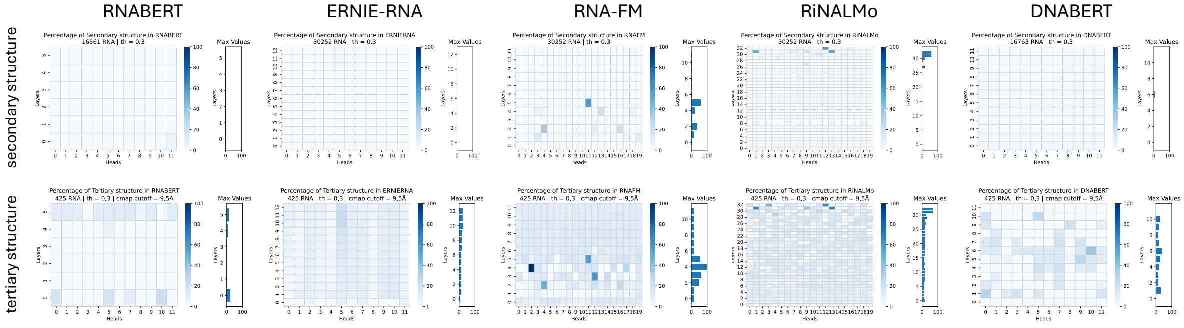

Figure S11. Evaluation for secondary (top row) and tertiary (bottom row) structure alignment using attention maps derived from nucleic acid LMs. In RNA-FM, the observed 100% alignment is actually an artifact seen when utilizing Equation 9 with thresholding (as explained in Figure S10).

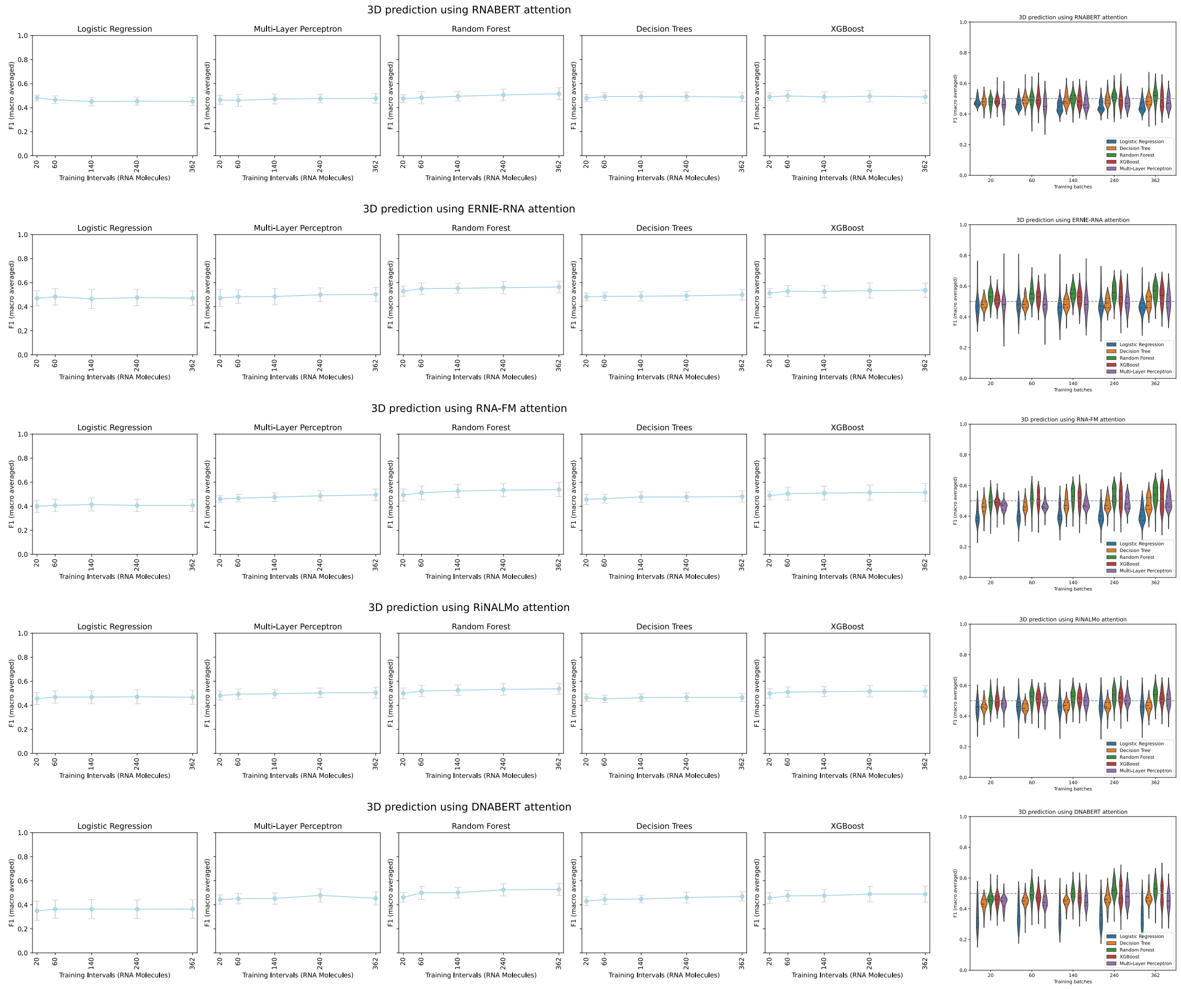

Figure S12. Changes in tertiary structure F_1 scores through training in five data batches of the initial training set in five classifiers utilizing features generated from the five nucleic acid LMs (RNABERT, ERNIE-RNA, RNA-FM, RiNALMo, DNABERT) attention. Data size did not improve the accuracy of the prediction for tertiary structure.

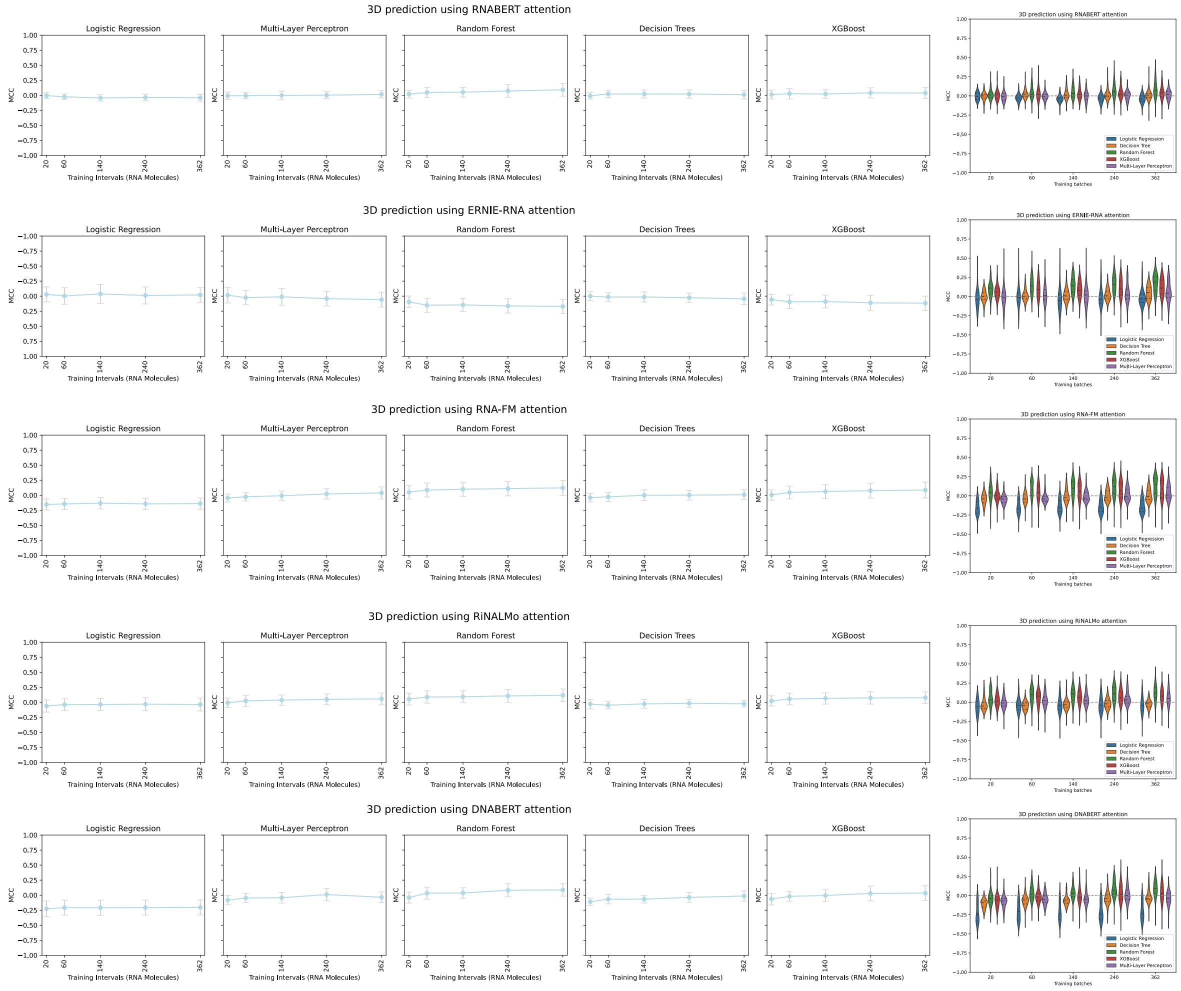

Figure S13. Changes in tertiary structure MCC scores through training in five data batches of the initial training set in five classifiers utilizing features generated from the five nucleic acid LMs (RNABERT, ERNIE-RNA, RNA-FM, RiNALMo, DNABERT) attention. MCC remained around 0 through training using any batch, which indicates no informative attention features and thus random predictions.

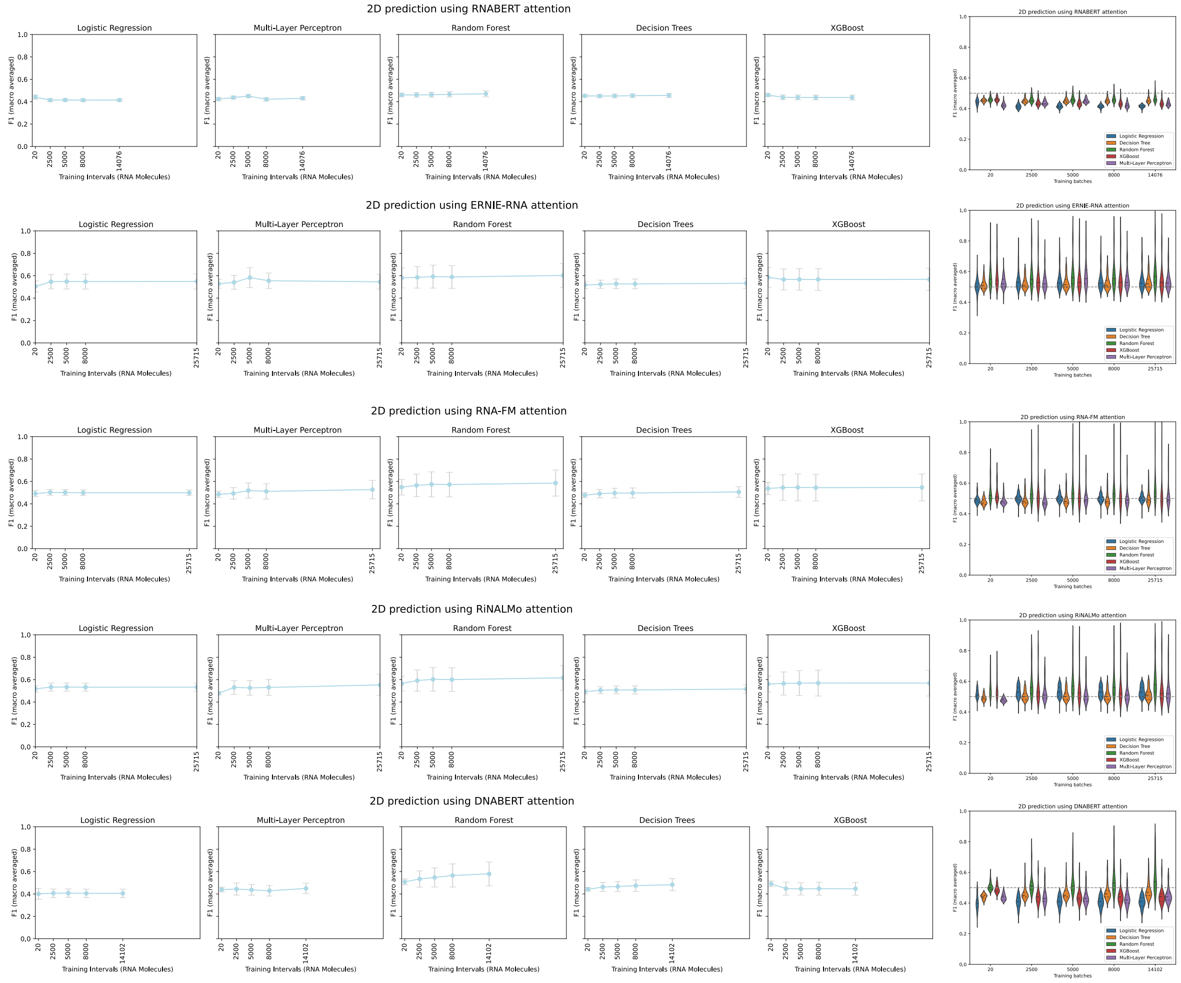

Figure S14. Changes in secondary structure F_1 scores through training in five data batches of the initial training set in five classifiers utilizing features generated from the five nucleic acid LMs (RNABERT, ERNIE-RNA, RNA-FM, RiNALMo, DNABERT) attention. The scores remained consistent throughout the training with each batch, with only the random forest showing a slight incremental improvement while being trained on more data from the training set.

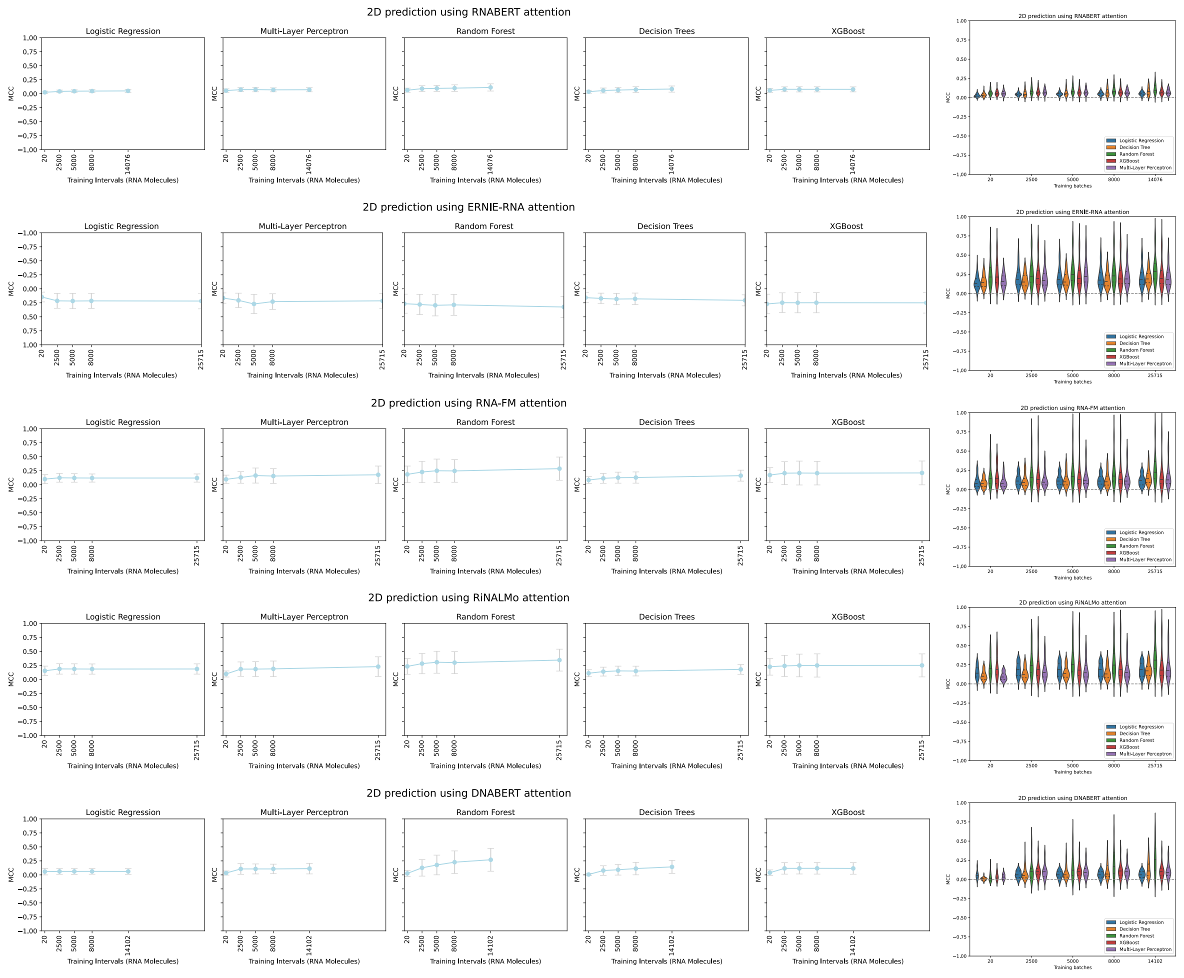

Figure S15. Changes in secondary structure MCC scores through training in five data batches of the initial training set in five classifiers utilizing features generated from the five nucleic acid LMs (RNABERT, ERNIE-RNA, RNA-FM, RiNALMo, DNABERT) attention.

Table S4. 3D predictions evaluation using the Logistic Regression classifier trained on 20 proteins and pLM attention features (F_1 macro averaged).

Protein Language Model	F_1 Score
**ESM2 – 3B**	0.81
**ProtT5 – uniref50 XL**	0.79
**ProtT5 – BFD XL**	0.76

Table S5. F_1 macro averaged score of the 3D predictions per classifier and training batch using nucleic acid LM attention.

Language Model	Batch Size	Logistic Regression	Multilayer Perceptron	Random Forest	Decision Trees	XGBoost Classifier
**RNABERT**	**20**	0.48	0.46	0.48	0.48	0.49
**ERNIE-RNA**		0.47	0.47	0.53	0.48	0.51
**RNA-FM**		0.40	0.46	0.49	0.46	0.49
**RiNALMo**		0.46	0.48	0.50	0.46	0.50
**DNABERT**		0.35	0.44	0.46	0.43	0.46
**RNABERT**	**60**	0.47	0.46	0.48	0.49	0.50
**ERNIE-RNA**		0.48	0.48	0.55	0.49	0.53
**RNA-FM**		0.41	0.47	0.51	0.46	0.50
**RiNALMo**		0.47	0.49	0.52	0.45	0.51
**DNABERT**		0.36	0.45	0.50	0.45	0.47
**RNABERT**	**140**	0.45	0.47	0.49	0.49	0.49
**ERNIE-RNA**		0.47	0.48	0.55	0.49	0.53
**RNA-FM**		0.41	0.47	0.53	0.48	0.51
**RiNALMo**		0.47	0.50	0.53	0.46	0.51
**DNABERT**		0.36	0.45	0.50	0.45	0.48
**RNABERT**	**240**	0.45	0.48	0.51	0.49	0.49
**ERNIE-RNA**		0.48	0.50	0.56	0.49	0.53
**RNA-FM**		0.41	0.49	0.53	0.48	0.51
**RiNALMo**		0.47	0.50	0.53	0.47	0.52
**DNABERT**		0.36	0.48	0.52	0.46	0.49
**RNABERT**	**362**	0.45	0.48	0.52	0.49	0.49
**ERNIE-RNA**		0.47	0.50	0.56	0.50	0.54
**RNA-FM**		0.41	0.49	0.54	0.48	0.51
**RiNALMo**		0.47	0.50	0.54	0.46	0.52
**DNABERT**		0.36	0.45	0.53	0.47	0.49

Table S6. MCC score of the 3D predictions per classifier and training batch using nucleic acid LM attention.

Language Model	Batch Size	Logistic Regression	Multilayer Perceptron	Random Forest	Decision Trees	XGBoost Classifier
**RNABERT**	**20**	-0.00	-0.01	0.02	-0.01	0.01
**ERNIE-RNA**		-0.03	-0.02	0.10	0.00	0.06
**RNA-FM**		-0.15	-0.05	0.05	-0.04	0.00
**RiNALMo**		-0.06	-0.01	0.05	-0.03	0.02
**DNABERT**		-0.23	-0.08	-0.04	-0.11	-0.07
**RNABERT**	**60**	-0.03	-0.01	0.05	0.02	0.03
**ERNIE-RNA**		-0.00	0.03	0.15	0.01	0.09
**RNA-FM**		-0.14	-0.03	0.09	-0.03	0.05
**RiNALMo**		-0.04	0.02	0.09	-0.05	0.05
**DNABERT**		-0.21	-0.05	0.03	-0.07	-0.02
**RNABERT**	**140**	-0.04	-0.00	0.05	0.02	0.02
**ERNIE-RNA**		-0.04	0.01	0.15	0.01	0.09
**RNA-FM**		-0.13	-0.01	0.10	-0.00	0.06
**RiNALMo**		-0.04	0.04	0.09	-0.03	0.07
**DNABERT**		-0.21	-0.04	0.04	-0.07	-0.01
**RNABERT**	**240**	-0.04	0.00	0.07	0.02	0.04
**ERNIE-RNA**		-0.01	0.04	0.16	0.03	0.11
**RNA-FM**		-0.14	0.02	0.11	0.00	0.08
**RiNALMo**		-0.03	0.05	0.11	-0.02	0.07
**DNABERT**		-0.21	0.01	0.08	-0.04	0.03
**RNABERT**	**362**	-0.04	0.01	0.09	0.01	0.04
**ERNIE-RNA**		-0.02	0.06	0.17	0.04	0.12
**RNA-FM**		-0.14	0.04	0.12	0.01	0.09
**RiNALMo**		-0.04	0.06	0.12	-0.02	0.08
**DNABERT**		-0.20	-0.04	0.09	-0.01	0.03

Table S7. 2D predictions evaluation per classifier and training batch using nucleic acid LM attention (F_1 macro averaged).

Language Model	Batch Size	Logistic Regression	Multilayer Perceptron	Random Forest	Decision Trees	XGBoost Classifier
RNABERT	20	0.44	0.42	0.46	0.45	0.46
ERNIE-RNA		0.5	0.53	0.58	0.52	0.59
RNA-FM		0.49	0.49	0.55	0.48	0.54
RiNALMo		0.52	0.48	0.57	0.49	0.56
DNABERT		0.4	0.44	0.51	0.44	0.49
RNABERT	2500	0.41	0.44	0.46	0.45	0.44
ERNIE-RNA		0.55	0.54	0.59	0.52	0.57
RNA-FM		0.5	0.49	0.57	0.49	0.55
RiNALMo		0.53	0.53	0.59	0.5	0.57
DNABERT		0.41	0.44	0.53	0.46	0.45
RNABERT	5000	0.41	0.45	0.46	0.45	0.44
ERNIE-RNA		0.55	0.58	0.59	0.53	0.57
RNA-FM		0.5	0.52	0.57	0.5	0.55
RiNALMo		0.53	0.53	0.6	0.51	0.57
DNABERT		0.41	0.44	0.55	0.47	0.45
RNABERT	8000	0.41	0.42	0.47	0.45	0.44
ERNIE-RNA		0.55	0.55	0.59	0.53	0.57
RNA-FM		0.50	0.51	0.57	0.5	0.54
RiNALMo		0.53	0.53	0.60	0.51	0.57
DNABERT		0.41	0.43	0.57	0.47	0.45
RNABERT	14076	0.41	0.43	0.47	0.46	0.44
ERNIE-RNA	25715	0.55	0.55	0.60	0.53	0.57
RNA-FM		0.50	0.53	0.59	0.51	0.55
RiNALMo		0.53	0.55	0.62	0.52	0.57
DNABERT	14102	0.41	0.45	0.58	0.48	0.45

Table S8. 2D predictions evaluation per classifier and training batch using nucleic acid LM attention (F_1 macro averaged).

Language Model	Batch Size	Logistic Regression	Multilayer Perceptron	Random Forest	Decision Trees	XGBoost Classifier
**RNABERT**	**20**	0.03	0.06	0.06	0.04	0.06
**ERNIE-RNA**		0.15	0.16	0.27	0.16	0.27
**RNA-FM**		0.1	0.1	0.19	0.09	0.17
**RiNALMo**		0.15	0.1	0.23	0.11	0.23
**DNABERT**		0.06	0.03	0.03	0.01	0.04
**RNABERT**	**2500**	0.04	0.07	0.09	0.06	0.08
**ERNIE-RNA**		0.21	0.21	0.28	0.17	0.25
**RNA-FM**		0.13	0.13	0.23	0.12	0.21
**RiNALMo**		0.19	0.18	0.28	0.14	0.24
**DNABERT**		0.06	0.11	0.13	0.08	0.12
**RNABERT**	**5000**	0.05	0.07	0.1	0.07	0.08
**ERNIE-RNA**		0.22	0.27	0.29	0.18	0.25
**RNA-FM**		0.12	0.17	0.25	0.13	0.21
**RiNALMo**		0.19	0.18	0.31	0.15	0.25
**DNABERT**		0.06	0.11	0.18	0.09	0.12
**RNABERT**	**8000**	0.05	0.07	0.1	0.07	0.08
**ERNIE-RNA**		0.22	0.23	0.29	0.18	0.25
**RNA-FM**		0.12	0.16	0.25	0.13	0.21
**RiNALMo**		0.18	0.19	0.30	0.15	0.25
**DNABERT**		0.06	0.11	0.23	0.11	0.12
**RNABERT**	**14076**	0.05	0.07	0.11	0.08	0.08
**ERNIE-RNA**	**25715**	0.22	0.21	0.32	0.21	0.25
**RNA-FM**		0.12	0.18	0.29	0.16	0.21
**RiNALMo**		0.19	0.23	0.35	0.18	0.25
**DNABERT**	**14102**	0.06	0.11	0.27	0.14	0.12

Table S9. 2D predictions evaluation of CNN using nucleic acid LM attention (F_1 macro averaged).

Language Model	Batch Size	F_1 Score
**RNABERT**	**14076**	0.51
**ERNIE-RNA**		0.51
**RNA-FM**	**25715**	0.51
**RiNALMo**		0.54
**DNABERT**	**14102**	0.50

Table S10. Evaluation of state-of-the-art 3D prediction tools (33 molecules - RMSD score).

PDB code	trRosettaRNA	AlphaFold3	RhoFold	DeepFoldRNA	FarFar2/ARES
7KUB	1.9	5.0	9.6	7.6	7.0
7KUC	1.6	2.6	1.7	1.5	1.4
7KUD	2.6	1.8	2.5	3.5	1.5
7MKT+	17.8	11.0	10.7	25.5	11.1
7PS8+	2.8	12.5	17.3	11.7	11.4
7Q48+	2.0	13.5	12.7	16.7	13.0
7RWR	2.2	6.4	5.1	5.8	9.0
7SHX	14.9	23.6	26.1	22.2	21.1
7UCR	1.0	0.4	1.7	1.1	5.5
7UMC	3.9	9.0	7.0	8.1	19.5
7UMD	2.3	6.7	6.2	8.7	10.0
7UME	2.5	2.0	2.9	2.8	2.6
7UR5	4.4	4.4	3.5	4.2	19.7
7UZ0	1.9	1.6	13.0	5.9	25.5
7V06	3.2	4.8	5.7	8.8	4.1
7WIA	1.7	10.6	5.8	4.8	7.2
8BWT	1.6	3.5	3.0	4.1	2.6
8CLR	1.9	1.4	1.9	9.8	1.4
8CQ1	2.8	3.2	5.2	2.9	4.3
8D28	1.1	4.8	2.8	3.1	5.2
8FB3	1.4	3.8	1.6	1.3	14.8
8FCS	2.9	3.3	6.6	3.3	5.5
8FZA	1.2	2.4	2.9	3.8	13.5
8HB8	2.2	12.2	14.1	12.8	18.3
8I44	4.4	4.2	5.3	4.5	4.7
8ITS	14.8	13.6	4.8	6.1	15.5
8JHP	4.6	4.7	3.3	3.7	10.2
8Q4O+	17.9	12.4	18.9	19.7	12.6
8SCF	3.1	3.0	3.0	2.5	2.9
8SCH	8.6	9.6	7.2	11.2	10.0
8THV	4.0	3.4	4.7	4.7	5.3
8TNS+	18.6	11.1	12.4	23.3	11.0
8UPT	4.3	3.7	4.2	3.6	12.1
**AVG**	**5.0**	**6.6**	**7.1**	**7.9**	**9.7**

Table S11. Evaluation of state-of-the-art 3D prediction tools (33 molecules - TM score).

PDB code	trRosettaRNA	AlphaFold3	RhoFold	DeepFoldRNA	FarFar2/ARES
7KUB	0.64132	0.43149	0.36741	0.3967	0.34462
7KUC	0.42389	0.34639	0.26534	0.46375	0.34414
7KUD	0.21656	0.36397	0.20742	0.35535	0.22994
7MKT+	0.10467	0.0672	0.03914	0.06757	0.07723
7PS8+	0.17924	0.08846	0.09645	0.10339	0.11792
7Q48+	0.25308	0.15402	0.11578	0.13651	0.07299
7RWR	0.44021	0.38511	0.33204	0.40155	0.28927
7SHX	0.2293	0.20458	0.18114	0.23269	0.26401
7UCR	0.49164	0.86516	0.31361	0.56398	0.28885
7UMC	0.67926	0.34146	0.31802	0.34274	0.28362
7UMD	0.41477	0.26432	0.24023	0.19604	0.26226
7UME	0.39621	0.33843	0.23238	0.33729	0.28627
7UR5	0.54984	0.58901	0.60241	0.63531	0.27064
7UZ0	0.80052	0.83226	0.43686	0.76093	0.26679
7V06	0.42163	0.32045	0.37522	0.38734	0.29002
7WIA	0.64047	0.38021	0.34183	0.37237	0.3967
8BWT	0.30712	0.21671	0.224	0.21151	0.30488
8CLR	0.21922	0.24664	0.24039	0.21917	0.28924
8CQ1	0.46238	0.35735	0.24848	0.42849	0.33071
8D28	0.57908	0.32646	0.22754	0.31261	0.30321
8FB3	0.4624	0.39384	0.401	0.57174	0.22749
8FCS	0.69476	0.72777	0.51315	0.60837	0.42549
8FZA	0.44185	0.22296	0.23458	0.20781	0.15768
8HB8	0.6842	0.30372	0.25725	0.1849	0.19615
8I44	0.14714	0.19657	0.1614	0.16265	0.19702
8ITS	0.22867	0.32981	0.30335	0.30556	0.29385
8JHP	0.17257	0.19149	0.21562	0.18974	0.18888
8Q4O+	0.14673	0.13847	0.11217	0.13977	0.11467
8SCF	0.38055	0.34102	0.25567	0.46268	0.30766
8SCH	0.28457	0.32328	0.36468	0.31473	0.23491
8THV	0.30614	0.28332	0.21762	0.23082	0.253
8TNS+	0.112	0.12154	0.11883	0.09244	0.12438
8UPT	0.53363	0.60048	0.50457	0.58214	0.32145
**AVG**	**0.39532**	**0.34224**	**0.27472**	**0.33269**	**0.25321**

Table S12. Comparison of the state-of-the-art 3D prediction tools with nucleic acid LM-RF (trained on Full dataset) (F_1 scores).

PDB code	trRosettaRNA	RhoFold	AlphaFlod3	DeepFoldRNA	FarFar2/ARES	RiNALMo-RF	RNA-FM-RF	ERNIE-RNA-RF	RNABERT-RF
7KUB	0.91	0.84	0.86	0.78	0.91	0.72	0.72	0.73	0.79
7KUC	0.86	0.87	0.94	0.90	0.97	0.56	0.60	0.61	0.67
7KUD	0.90	0.81	0.92	0.83	0.90	0.58	0.59	0.62	0.65
7MKT$^+$	0.51	0.54	0.62	0.44	0.60	0.56	0.59	0.60	0.76
7PS8$^+$	0.88	0.48	0.49	0.58	0.55	0.44	0.46	0.41	0.39
7Q48$^+$	0.94	0.51	0.50	0.52	0.55	0.40	0.38	0.43	0.64
7RWR	0.86	0.84	0.75	0.78	0.85	0.68	0.69	0.71	0.77
7SHX	0.84	0.78	0.70	0.66	0.80	0.55	0.54	0.55	0.53
7UCR	0.91	0.93	0.96	0.92	0.88	0.48	0.60	0.59	0.46
7UMC	0.90	0.87	0.83	0.79	0.77	0.67	0.66	0.65	0.72
7UMD	0.89	0.87	0.84	0.71	0.82	0.51	0.52	0.56	0.47
7UME	0.89	0.89	0.86	0.81	0.87	0.64	0.66	0.67	0.74
7UR5	0.86	0.92	0.89	0.84	0.85	0.73	0.74	0.70	0.75
7UZ0	0.90	0.83	0.94	0.89	0.72	0.63	0.58	0.64	0.55
7V06	0.87	0.89	0.87	0.79	0.91	0.67	0.68	0.67	0.72
7WIA	0.91	0.89	0.87	0.86	0.87	0.67	0.70	0.66	0.73
8BWT	0.92	0.86	0.87	0.85	0.91	0.58	0.64	0.65	0.43
8CLR	0.90	0.93	0.88	0.82	0.93	0.53	0.50	0.57	0.50
8CQ1	0.87	0.87	0.82	0.83	0.89	0.51	0.59	0.59	0.55
8D28	0.92	0.87	0.80	0.89	0.85	0.67	0.68	0.70	0.76
8FB3	0.94	0.94	0.85	0.89	0.66	0.53	0.49	0.52	0.51
8FCS	0.89	0.9	0.91	0.85	0.92	0.60	0.60	0.62	0.52
8FZA	0.93	0.89	0.85	0.81	0.62	0.57	0.48	0.49	0.50
8HB8	0.93	0.77	0.76	0.68	0.66	0.49	0.50	0.51	0.47
8I44	0.89	0.91	0.90	0.87	0.89	0.57	0.58	0.62	0.54
8ITS	0.68	0.87	0.81	0.82	0.83	0.52	0.52	0.56	0.52
8JHP	0.82	0.87	0.79	0.83	0.73	0.56	0.63	0.63	0.41
8Q4O$^+$	0.49	0.59	0.53	0.50	0.60	0.42	0.40	0.42	0.45
8SCF	0.87	0.9	0.82	0.87	0.89	0.60	0.62	0.60	0.48
8SCH	0.82	0.86	0.86	0.81	0.89	0.59	0.59	0.61	0.51
8THV	0.82	0.85	0.83	0.81	0.82	0.57	0.59	0.59	0.51
8TNS$^+$	0.51	0.56	0.68	0.49	0.65	0.53	0.60	0.49	0.73
8UPT	0.89	0.91	0.91	0.86	0.87	0.65	0.59	0.64	0.55
**AVG**	**0.85**	**0.82**	**0.81**	**0.77**	**0.76**	**0.58**	**0.59**	**0.59**	**0.58**

Table S13. Comparison of the state-of-the-art 3D prediction tools with nucleic acid LM-RF (trained on Full dataset) (MCC scores).

PDB code	trRosettaRNA	RhoFold	AlphaFlod3	DeepFoldRNA	FarFar2/ARES	RiNALMo-RF	RNA-FM-RF	ERNIE-RNA-RF	RNABERT-RF
7KUB	0.89	0.68	0.83	0.73	0.83	0.45	0.45	0.46	0.59
7KUC	0.71	0.75	0.86	0.74	0.94	0.2	0.33	0.38	0.47
7KUD	0.76	0.63	0.8	0.55	0.8	0.37	0.42	0.45	0.45
7MKT+	0.41	0.36	0.25	0.36	0.22	0.2	0.3	0.32	0.62
7PS8+	0.66	0.32	0.2	0.24	0.2	0.01	0.12	0.05	0.02
7Q48+	0.86	0.06	0.04	0.38	0.12	-0.16	-0.17	-0.06	0.39
7RWR	0.8	0.68	0.66	0.69	0.7	0.37	0.39	0.43	0.56
7SHX	0.82	0.57	0.66	0.61	0.61	0.09	0.07	0.1	0.06
7UCR	0.87	0.87	0.94	0.87	0.76	0.12	0.22	0.25	-0.01
7UMC	0.88	0.74	0.8	0.74	0.54	0.34	0.32	0.31	0.43
7UMD	0.85	0.74	0.8	0.59	0.63	0.02	0.04	0.12	-0.03
7UME	0.83	0.78	0.78	0.7	0.74	0.28	0.33	0.35	0.5
7UR5	0.84	0.84	0.87	0.81	0.7	0.47	0.48	0.41	0.49
7UZ0	0.89	0.66	0.93	0.87	0.43	0.28	0.17	0.28	0.1
7V06	0.82	0.79	0.83	0.71	0.83	0.34	0.36	0.35	0.45
7WIA	0.88	0.79	0.83	0.82	0.75	0.35	0.41	0.32	0.46
8BWT	0.85	0.74	0.78	0.74	0.83	0.18	0.32	0.38	0.1
8CLR	0.76	0.87	0.73	0.59	0.87	0.24	0.24	0.34	0.29
8CQ1	0.83	0.75	0.76	0.78	0.78	0.03	0.18	0.18	0.1
8D28	0.88	0.75	0.7	0.83	0.7	0.36	0.37	0.41	0.54
8FB3	0.89	0.87	0.75	0.82	0.34	0.07	-0.01	0.07	0.08
8FCS	0.86	0.8	0.89	0.81	0.84	0.2	0.21	0.25	0.05
8FZA	0.87	0.78	0.72	0.66	0.25	0.15	-0.02	0.05	0.15
8HB8	0.9	0.54	0.69	0.59	0.32	-0.01	0.02	0.02	-0.01
8I44	0.81	0.83	0.84	0.79	0.79	0.16	0.18	0.27	0.11
8ITS	0.57	0.75	0.75	0.75	0.66	0.05	0.05	0.13	0.08
8JHP	0.69	0.75	0.68	0.71	0.47	0.14	0.3	0.3	-0.06
8Q4O+	0.36	0.38	0.22	0.36	0.22	-0.12	-0.13	-0.06	0.02
8SCF	0.8	0.82	0.76	0.8	0.79	0.2	0.26	0.22	0.08
8SCH	0.79	0.73	0.84	0.78	0.78	0.18	0.18	0.22	0.02
8THV	0.74	0.72	0.74	0.68	0.64	0.15	0.2	0.21	0.1
8TNS+	0.41	0.38	0.37	0.41	0.33	0.08	0.3	0.08	0.51
8UPT	0.87	0.82	0.89	0.83	0.74	0.34	0.22	0.29	0.1
**AVG**	**0.78**	**0.68**	**0.70**	**0.68**	**0.61**	**0.19**	**0.22**	**0.24**	**0.23**

Table S14. Comparison of the state-of-the-art 2D prediction tools with nucleic acid LM-RF (trained on Full dataset) (F_1 scores).

PDB code	SpotRNA	RNAFold	mxFold2	RNA-MSM	RiNALMo-RF	RNA-FM-RF	ERNIE-RNA-RF	RNABERT-RF
7KUB	1.0	1.0	0.68	0.90	0.63	0.71	0.65	0.49
7KUC	1.0	1.0	0.49	1.0	0.74	0.77	1.0	0.62
7KUD	0.94	0.94	0.49	1.0	0.78	1.0	0.71	0.63
7MKT$^+$	1.0	0.50	0.50	1.0	0.48	1.0	0.50	0.47
7PS8$^+$	0.5	0.50	0.50	0.50	0.50	0.49	0.49	0.46
7Q48$^+$	0.5	0.49	0.50	0.50	0.49	0.49	0.50	0.46
7RWR	0.83	0.93	0.90	0.88	0.73	0.76	0.71	0.53
7SHX	0.79	0.79	0.60	0.88	0.56	0.62	0.61	0.47
7UCR	0.88	0.93	0.77	0.90	0.74	0.77	0.83	0.53
7UMC	0.83	0.80	0.73	0.93	0.61	0.62	0.65	0.50
7UMD	0.82	1.0	0.83	0.92	0.71	0.76	0.76	0.52
7UME	1.0	1.0	0.83	0.92	0.78	0.79	0.80	0.57
7UR5	0.98	0.93	0.93	0.94	0.68	0.71	0.70	0.49
7UZ0	0.77	0.99	0.91	0.91	0.67	0.77	0.72	0.48
7V06	0.98	0.98	0.78	0.96	0.72	0.73	0.72	0.51
7WIA	1.0	0.98	0.83	0.97	0.67	0.75	0.72	0.51
8BWT	1.0	1.0	0.93	0.92	0.78	0.81	0.78	0.57
8CLR	0.95	0.95	0.48	1.0	0.95	1.0	1.0	0.68
8CQ1	1.0	1.0	0.72	0.99	0.69	0.63	0.74	0.52
8D28	0.97	0.97	0.91	0.91	0.75	0.77	0.73	0.52
8FB3	0.95	0.95	0.49	0.95	0.66	0.75	0.68	0.50
8FCS	1.0	1.0	0.88	0.93	0.68	0.83	0.69	0.51
8FZA	0.49	0.83	0.50	0.97	0.70	0.74	0.71	0.49
8HB8	0.63	0.72	0.63	0.92	0.60	0.62	0.63	0.50
8I44	0.93	0.93	0.49	0.90	0.83	0.81	0.84	0.60
8ITS	0.98	0.98	0.84	0.94	0.74	0.84	0.77	0.52
8JHP	0.73	0.97	0.49	0.91	0.70	0.75	0.72	0.54
8Q4O$^+$	0.50	0.50	1.0	0.50	0.50	0.49	0.49	0.45
8SCF	0.97	0.97	0.81	0.95	0.70	0.79	0.71	0.53
8SCH	0.97	0.97	0.86	0.97	0.65	0.70	0.64	0.50
8THV	0.95	0.95	0.73	0.98	0.73	0.80	0.78	0.57
8TNS$^+$	1.0	0.50	0.50	1.0	0.48	0.50	0.49	0.47
8UPT	0.85	0.72	0.82	0.83	0.62	0.71	0.62	0.46
**AVG**	**0.90**	**0.87**	**0.87**	**0.71**	**0.74**	**0.67**	**0.70**	**0.52**

Table S15. Comparison of the state-of-the-art 2D prediction tools with nucleic acid LM-RF (trained on Full dataset) (MCC scores).

PDB code	SpotRNA	RNAFold	mxFold2	RNA-MSM	RiNALMo-RF	RNA-FM-RF	ERNIE-RNA-RF	RNABERT-RF
7KUB	0.81	1.0	1.0	0.39	0.49	0.38	0.41	0.14
7KUC	1.0	1.0	1.0	-0.03	0.58	0.48	1.0	0.41
7KUD	1.0	0.89	0.89	0.0	1.0	0.56	0.52	0.43
7MKT+	0.0	0.0	0.0	0.0	0.0	0.0	0.0	0.0
7PS8+	0.0	0.0	0.0	0.0	0.0	0.0	0.0	0.0
7Q48+	0.0	0.0	0.0	0.0	0.0	0.0	0.0	0.0
7RWR	0.77	0.66	0.87	0.80	0.57	0.53	0.53	0.24
7SHX	0.76	0.58	0.59	0.22	0.36	0.25	0.33	0.14
7UCR	0.81	0.77	0.86	0.58	0.60	0.51	0.70	0.22
7UMC	0.86	0.66	0.6	0.51	0.34	0.35	0.38	0.13
7UMD	0.85	0.64	1.0	0.70	0.55	0.48	0.59	0.15
7UME	0.84	1.0	1.0	0.67	0.62	0.57	0.64	0.30
7UR5	0.88	0.97	0.87	0.86	0.48	0.43	0.51	0.17
7UZ0	0.83	0.53	0.98	0.83	0.61	0.45	0.54	0.16
7V06	0.93	0.97	0.97	0.63	0.52	0.53	0.52	0.18
7WIA	0.94	1.0	0.97	0.69	0.58	0.42	0.53	0.22
8BWT	0.85	1.0	1.0	0.86	0.68	0.58	0.62	0.24
8CLR	1.0	0.91	0.91	0.0	1.0	0.91	1.0	0.49
8CQ1	0.97	1.0	1.0	0.54	0.29	0.45	0.57	0.21
8D28	0.83	0.95	0.95	0.83	0.6	0.56	0.56	0.22
8FB3	0.91	0.91	0.91	-0.01	0.51	0.42	0.44	0.12
8FCS	0.86	1.0	1.0	0.77	0.69	0.45	0.48	0.21
8FZA	0.93	-0.01	0.67	-0.01	0.51	0.43	0.46	0.05
8HB8	0.83	0.26	0.43	0.39	0.33	0.3	0.31	0.09
8I44	0.79	0.85	0.85	0.0	0.64	0.65	0.72	0.34
8ITS	0.89	0.97	0.97	0.69	0.71	0.57	0.61	0.20
8JHP	0.82	0.46	0.93	0.0	0.53	0.46	0.54	0.14
8Q4O+	0.0	0.0	0.0	0.0	0.0	0.0	0.0	0.0
8SCF	0.90	0.95	0.95	0.62	0.65	0.41	0.53	0.14
8SCH	0.93	0.93	0.93	0.73	0.49	0.43	0.41	0.19
8THV	0.95	0.90	0.90	0.47	0.62	0.51	0.61	0.33
8TNS+	0.0	0.0	0.0	0.0	0.0	0.0	0.0	0.0
8UPT	0.68	0.72	0.44	0.68	0.52	0.35	0.38	0.12
**AVG**	**0.74**	**0.68**	**0.75**	**0.41**	**0.49**	**0.41**	**0.47**	**0.18**

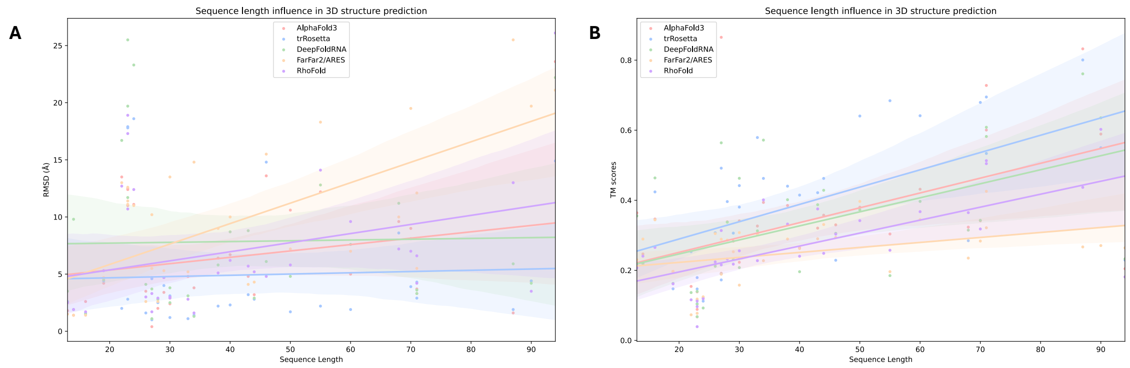

Figure S16. Sequence length appears to influence the 3D structure prediction of RNA, as observed while using: **A**. RMSD **B**. TM score.

References

1. Zheng,W., Li,Y., Zhang,C., Pearce,R., Mortuza,S.M. and Zhang,Y. (2019) Deep-learning contact-map guided protein structure prediction in CASP13. *Proteins*, **87**, 1149–1164.
2. Lawson,C.L., Berman,H.M., Chen,L., Vallat,B. and Zirbel,C.L. (2024) The Nucleic Acid Knowledgebase: a new portal for 3D structural information about nucleic acids. *Nucleic Acids Res*, **52**, D245–D254.
3. Danaee,P., Rouches,M., Wiley,M., Deng,D., Huang,L. and Hendrix,D. (2018) bpRNA: large-scale automated annotation and analysis of RNA secondary structure. *Nucleic Acids Res*, **46**, 5381–5394.
4. Tan,Z., Fu,Y., Sharma,G. and Mathews,D.H. (2017) TurboFold II: RNA structural alignment and secondary structure prediction informed by multiple homologs. *Nucleic Acids Res*, **45**, 11570.
5. Sloma,M.F. and Mathews,D.H. (2016) Exact calculation of loop formation probability identifies folding motifs in RNA secondary structures. *RNA*, **22**, 1808–1818.
6. Fu,L., Niu,B., Zhu,Z., Wu,S. and Li,W. (2012) CD-HIT: accelerated for clustering the next-generation sequencing data. *Bioinformatics*, **28**, 3150.
7. Cock,P.J.A., Antao,T., Chang,J.T., Chapman,B.A., Cox,C.J., Dalke,A., Friedberg,I., Hamelryck,T., Kauff,F., Wilczynski,B., *et al.* (2009) Biopython: freely available Python tools for computational molecular biology and bioinformatics. *Bioinformatics*, **25**, 1422–1423.
8. Lin,Z., Akin,H., Rao,R., Hie,B., Zhu,Z., Lu,W., Smetanin,N., Verkuil,R., Kabeli,O., Shmueli,Y., *et al.* (2023) Evolutionary-scale prediction of atomic-level protein structure with a language model. *Science (1979)*, **379**, 1123–1130.
9. Elnaggar,A., Heinzinger,M., Dallago,C., Rehawi,G., Wang,Y., Jones,L., Gibbs,T., Feher,T., Angerer,C., Steinegger,M., *et al.* (2021) ProtTrans: Towards Cracking the Language of Life's Code Through Self-Supervised Learning. *bioRxiv*, **14**, 2020.07.12.199554.
10. Suzek,B.E., Wang,Y., Huang,H., McGarvey,P.B. and Wu,C.H. (2015) UniRef clusters: a comprehensive and scalable alternative for improving sequence similarity searches. *Bioinformatics*, **31**, 926.
11. Steinegger,M. and Söding,J. (2018) Clustering huge protein sequence sets in linear time. *Nature Communications 2018 9:1*, **9**, 1–8.
12. Steinegger,M., Mirdita,M. and Söding,J. (2019) Protein-level assembly increases protein sequence recovery from metagenomic samples manyfold. *Nature Methods 2019 16:7*, **16**, 603–606.
13. Akiyama,M. and Sakakibara,Y. (2022) Informative RNA base embedding for RNA structural alignment and clustering by deep representation learning. *NAR Genom Bioinform*, **4**.
14. Chen,J., Hu,Z., Sun,S., Tan,Q., Wang,Y., Yu,Q., Zong,L., Hong,L., Xiao,J., Shen,T., *et al.* (2022) Interpretable RNA Foundation Model from Unannotated Data for Highly Accurate RNA Structure and Function Predictions. *bioRxiv*, 10.1101/2022.08.06.503062.

15. Wang,N., Bian,J., Li,Y., Li,X., Mumtaz,S., Kong,L. and Xiong,H. (2024) Multi-purpose RNA language modelling with motif-aware pretraining and type-guided fine-tuning. *Nature Machine Intelligence 2024 6:5*, **6**, 548–557.
16. Josip,R., Peni´c,P., Vlaši´c,T.V., Huber,R.G., Wan,Y. and Siki´c,M.S. (2024) RiNALMo: General-Purpose RNA Language Models Can Generalize Well on Structure Prediction Tasks.
17. Ji,Y., Zhou,Z., Liu,H. and Davuluri,R. V. (2021) DNABERT: pre-trained Bidirectional Encoder Representations from Transformers model for DNA-language in genome. *Bioinformatics*, **37**, 2112–2120.
18. Dunn,S.D., Wahl,L.M. and Gloor,G.B. (2008) Mutual information without the influence of phylogeny or entropy dramatically improves residue contact prediction. *Bioinformatics*, **24**, 333–340.
19. Lemaˆıtre Lemaˆıtre,G., Nogueira,F. and Aridas char,C.K. (2017) Imbalanced-learn: A Python Toolbox to Tackle the Curse of Imbalanced Datasets in Machine Learning. *Journal of Machine Learning Research*, **18**, 1–5.
20. Cox,D.R. (1958) The Regression Analysis of Binary Sequences. *J R Stat Soc Series B Stat Methodol*, **20**, 215–232.
21. Quinlan,J.R. (1986) Induction of Decision Trees. *Mach Learn*, **1**, 81–106.
22. Ho,T.K. (1995) Random decision forests. *Proceedings of the International Conference on Document Analysis and Recognition, ICDAR*, **1**, 278–282.
23. Lecun,Y., Bengio,Y. and Hinton,G. (2015) Deep learning. *Nature 2015 521:7553*, **521**, 436–444.
24. Pedregosa FABIANPEDREGOSA,F., Michel,V., Grisel OLIVIERGRISEL,O., Blondel,M., Prettenhofer,P., Weiss,R., Vanderplas,J., Cournapeau,D., Pedregosa,F., Varoquaux,G., *et al.* (2011) Scikit-learn: Machine Learning in Python. *Journal of Machine Learning Research*, **12**, 2825–2830.
25. Chen,T. and Guestrin,C. XGBoost: A Scalable Tree Boosting System. *Proceedings of the 22nd ACM SIGKDD International Conference on Knowledge Discovery and Data Mining*, 10.1145/2939672.
26. Lecun,Y., Bengio,Y. and 4g332,R. Convolutional Networks for Images, Speech, and Time-Series.
27. Paszke,A., Gross,S., Massa,F., Lerer,A., Bradbury,J., Chanan,G., Killeen,T., Lin,Z., Gimelshein,N., Antiga,L., *et al.* (2019) PyTorch: An Imperative Style, High-Performance Deep Learning Library. *Adv Neural Inf Process Syst*, **32**.
28. Wang,W., Feng,C., Han,R., Wang,Z., Ye,L., Du,Z., Wei,H., Zhang,F., Peng,Z. and Yang,J. (2023) trRosettaRNA: automated prediction of RNA 3D structure with transformer network. *Nature Communications 2023 14:1*, **14**, 1–13.
29. Abramson,J., Adler,J., Dunger,J., Evans,R., Green,T., Pritzel,A., Ronneberger,O., Willmore,L., Ballard,A.J., Bambrick,J., *et al.* (2024) Accurate structure prediction of biomolecular interactions with AlphaFold 3. *Nature 2024 630:8016*, **630**, 493–500.
30. Pearce,R., Omenn,G.S. and Zhang,Y. (2022) De Novo RNA Tertiary Structure Prediction at Atomic Resolution Using Geometric Potentials from Deep Learning. *bioRxiv*, 10.1101/2022.05.15.491755.
31. Watkins,A.M., Rangan,R. and Das,R. (2020) FARFAR2: Improved De Novo Rosetta Prediction of Complex Global RNA Folds. *Structure*, **28**, 963-976.e6.
32. Townshend,R.J.L., Eismann,S., Watkins,A.M., Rangan,R., Karelina,M., Das,R. and Dror,R.O. (2021) Geometric deep learning of RNA structure. *Science (1979)*, **373**, 1047–1051.

33. Singh,J., Hanson,J., Paliwal,K. and Zhou,Y. (2019) RNA secondary structure prediction using an ensemble of two-dimensional deep neural networks and transfer learning. *Nature Communications 2019 10:1*, **10**, 1–13.
34. Lorenz,R., Bernhart,S.H., Höner zu Siederdissen,C., Tafer,H., Flamm,C., Stadler,P.F. and Hofacker,I.L. (2011) ViennaRNA Package 2.0. *Algorithms for Molecular Biology*, **6**, 1–14.
35. Sato,K., Akiyama,M. and Sakakibara,Y. (2021) RNA secondary structure prediction using deep learning with thermodynamic integration. *Nature Communications 2021 12:1*, **12**, 1–9.
36. Zhang,Y., Lang,M., Jiang,J., Gao,Z., Xu,F., Litfin,T., Chen,K., Singh,J., Huang,X., Song,G., *et al.* (2024) Multiple sequence alignment-based RNA language model and its application to structural inference. *Nucleic Acids Res*, **52**, e3–e3.
37. Parisien,M., Cruz,J.A., Westhof,É. and Major,F. (2009) New metrics for comparing and assessing discrepancies between RNA 3D structures and models. *RNA*, **15**, 1875.
38. Wagner,N. (2019) Stop Codon Readthrough in VEGF-a Is Regulated by Synergistic Activity of a Complex Tripartite Signal.
39. Roschdi,S., Yan,J., Nomura,Y., Escobar,C.A., Petersen,R.J., Bingman,C.A., Tonelli,M., Vivek,R., Montemayor,E.J., Wickens,M., *et al.* (2022) An atypical RNA quadruplex marks RNAs as vectors for gene silencing. *Nat Struct Mol Biol*, **29**, 1113–1121.
40. Berman,H.M., Westbrook,J., Feng,Z., Gilliland,G., Bhat,T.N., Weissig,H., Shindyalov,I.N. and Bourne,P.E. (2000) The Protein Data Bank. *Nucleic Acids Res*, **28**, 235–242.
41. Samuelian,J.S., Gremminger,T.J., Song,Z., Poudyal,R.R., Li,J., Zhou,Y., Staller,S.A., Carballo,J.A., Roychowdhury-Saha,M., Chen,S.J., *et al.* (2022) An RNA aptamer that shifts the reduction potential of metabolic cofactors. *Nat Chem Biol*, **18**, 1263–1269.
42. Sharma,S., Pisignano,G., Merulla,J., Catapano,C. V. and Varani,G. (2022) A functional SNP regulates E-cadherin expression by dynamically remodeling the 3D structure of a promoter-associated non-coding RNA transcript. *Nucleic Acids Res*, **50**, 11331–11343.
43. Harp,J.M., Lybrand,T.P., Pallan,P.S., Coates,L., Sullivan,B. and Egli,M. (2022) Cryo neutron crystallography demonstrates influence of RNA 2’-OH orientation on conformation, sugar pucker and water structure. *Nucleic Acids Res*, **50**, 7721–7738.
44. Sun,Y.T. and Varani,G. (2022) Structure of the dengue virus RNA promoter. *RNA*, **28**, 1210–1223.
45. Prabhakar,A., Krahn,N., Zhang,J., Vargas-Rodriguez,O., Krupkin,M., Fu,Z., Acosta-Reyes,F.J., Ge,X., Choi,J., Crnković,A., *et al.* (2022) Uncovering translation roadblocks during the development of a synthetic tRNA. *Nucleic Acids Res*, **50**, 10201–10211.
46. Grigg,J.C., Price,I.R. and Ke,A. (2022) tRNA Fusion to Streamline RNA Structure Determination: Case Studies in Probing Aminoacyl-tRNA Sensing Mechanisms by the T-Box Riboswitch. *Crystals (Basel)*, **12**, 694.
47. Chiu,L.Y., Emery,A., Jain,N., Sugarman,A., Kendrick,N., Luo,L., Ford,W., Swanstrom,R. and Tolbert,B.S. (2022) Encoded Conformational Dynamics of the HIV Splice Site A3 Regulatory Locus: Implications for Differential Binding of hnRNP Splicing Auxiliary Factors. *J Mol Biol*, **434**.
48. Xu,L., Xiao,Y., Zhang,J. and Fang,X. (2023) Structural insights into translation regulation by the THF-II riboswitch. *Nucleic Acids Res*, **51**, 952–965.
49. Vögele,J., Duchardt-Ferner,E., Bains,J.K., Knezic,B., Wacker,A., Sich,C., Weigand,J.E., Šponer,J., Schwalbe,H., Krepl,M., *et al.* (2024) Structure of an internal loop motif with three consecutive U•U mismatches from stem-loop 1 in the 3’-UTR of the SARS-CoV-2 genomic RNA. *Nucleic Acids Res*, **52**.

50. Oxenfarth,A., Kümmerer,F., Bottaro,S., Schnieders,R., Pinter,G., Jonker,H.R.A., Fürtig,B., Richter,C., Blackledge,M., Lindorff-Larsen,K., *et al.* (2023) Integrated NMR/Molecular Dynamics Determination of the Ensemble Conformation of a Thermodynamically Stable CUUG RNA Tetraloop. *J Am Chem Soc*, **145**, 16557–16572.
51. Vögele,J., Hymon,D., Martins,J., Ferner,J., Jonker,H.R.A., Hargrove,A.E., Weigand,J.E., Wacker,A., Schwalbe,H., Wöhnert,J., *et al.* (2023) High-resolution structure of stem-loop 4 from the 5′-UTR of SARS-CoV-2 solved by solution state NMR. *Nucleic Acids Res*, **51**, 11318–11331.
52. Menichelli,E., Lam,B.J., Wang,Y., Wang,V.S., Shaffer,J., Tjhung,K.F., Bursulaya,B., Nguyen,T.N., Vo,T., Alper,P.B., *et al.* (2022) Discovery of small molecules that target a tertiary-structured RNA. *Proc Natl Acad Sci U S A*, **119**.
53. Schroeder,G.M., Akinyemi,O., Malik,J., Focht,C.M., Pritchett,E.M., Baker,C.D., Mcsally,J.P., Jenkins,J.L., Mathews,D.H. and Wedekind,J.E. (2023) A riboswitch separated from its ribosome-binding site still regulates translation. *Nucleic Acids Res*, **51**, 2464–2484.
54. Ma,S., Kotar,A., Grote,S., Rouskin,S. and Keane,S.C. (2023) Structure of pre-miR-31 reveals an active role in Dicer processing. *bioRxiv*, 10.1101/2023.01.03.519659.
55. Schroeder,G.M., Kiliushik,D., Jenkins,J.L. and Wedekind,J.E. (2023) Structure and function analysis of a type III preQ1-I riboswitch from Escherichia coli reveals direct metabolite sensing by the Shine-Dalgarno sequence. *J Biol Chem*, **299**.
56. Peng,X., Liao,W., Lin,X., Lilley,D.M.J. and Huang,L. (2023) Crystal structures of the NAD+-II riboswitch reveal two distinct ligand-binding pockets. *Nucleic Acids Res*, **51**, 2904–2914.
57. Ichijo,R., Kamimura,T. and Kawai,G. (2023) Interaction between a fluoroquinolone derivative KG022 and RNAs: Effect of base pairs 3’ adjacent to the bulged residues. *Front Mol Biosci*, **10**.
58. RCSB PDB - 8ITS: Crystal structure of DUF-3268 k-junction.
59. RCSB PDB - 8JHP: Another hairpin structure found in the RNA element involved in piRNA biogenesis.
60. Orehova,M., Plavec,J. and Kocman,V. (2024) High-Resolution Structure of RNA G-Quadruplex Containing Unique Structural Motifs Originating from the 5′-UTR of Human Tyrosine Kinase 2 (TYK2). *ACS Omega*, **9**, 7215–7229.
61. Warden,M.S., Derose,E.F., Tamayo,J. V., Mueller,G.A., Gavis,E.R. and Hall,T.M.T. (2023) The translational repressor Glorund uses interchangeable RNA recognition domains to recognize Drosophila nanos. *Nucleic Acids Res*, **51**, 8836–8849.
62. Roy,R., Geng,A., Shi,H., Merriman,D.K., Dethoff,E.A., Salmon,L. and Al-Hashimi,H.M. (2023) Kinetic Resolution of the Atomic 3D Structures Formed by Ground and Excited Conformational States in an RNA Dynamic Ensemble. *J Am Chem Soc*, **145**, 22964–22978.
63. Escobar,C.A., Petersen,R.J., Tonelli,M., Fan,L., Henzler-Wildman,K.A. and Butcher,S.E. (2023) Solution Structure of Poly(UG) RNA. *J Mol Biol*, **435**.
64. Krahn,N., Zhang,J., Melnikov,S. V., Tharp,J.M., Villa,A., Patel,A., Howard,R.J., Gabir,H., Patel,T.R., Stetefeld,J., *et al.* (2024) tRNA shape is an identity element for an archaeal pyrrolysyl-tRNA synthetase from the human gut. *Nucleic Acids Res*, **52**, 513–524.
